# Favorable vaccine-induced SARS-CoV-2–specific T cell response profile in patients undergoing immune-modifying therapies

**DOI:** 10.1172/JCI159500

**Published:** 2022-06-15

**Authors:** Martin Qui, Nina Le Bert, Webber Pak Wo Chan, Malcolm Tan, Shou Kit Hang, Smrithi Hariharaputran, Jean Xiang Ying Sim, Jenny Guek Hong Low, Weiling Ng, Wei Yee Wan, Tiing Leong Ang, Antonio Bertoletti, Ennaliza Salazar

**Affiliations:** 1 Programme in Emerging Infectious Diseases, Duke-NUS Medical School, Singapore.; 2 Department of Gastroenterology and Hepatology,; 3 Department of Infectious Disease, and; 4 Department of Microbiology, Singapore General Hospital, Singapore.; 5 Department of Gastroenterology and Hepatology, Changi General Hospital, Singapore.; 6 Singapore Immunology Network, A*STAR; Singapore.

**Keywords:** COVID-19, Immunology, Cellular immune response, Immunotherapy, Inflammatory bowel disease

## Abstract

**BACKGROUND:**

Patients undergoing immune-modifying therapies demonstrate a reduced humoral response after COVID-19 vaccination, but we lack a proper evaluation of the effect of such therapies on vaccine-induced T cell responses.

**METHODS:**

We longitudinally characterized humoral and spike-specific T cell responses in patients with inflammatory bowel disease (IBD), who were on antimetabolite therapy (azathioprine or methotrexate), TNF inhibitors, and/or other biologic treatment (anti-integrin or anti-p40) for up to 6 months after completing 2-dose COVID-19 mRNA vaccination.

**RESULTS:**

We demonstrate that a spike-specific T cell response was not only induced in treated patients with IBD at levels similar to those of healthy individuals, but also sustained at higher magnitude for up to 6 months after vaccination, particularly in those treated with TNF inhibitor therapy. Furthermore, the spike-specific T cell response in these patients was mainly preserved against mutations present in SARS-CoV-2 B.1.1.529 (Omicron) and characterized by a Th1/IL-10 cytokine profile.

**CONCLUSION:**

Despite the humoral response defects, patients under immune-modifying therapies demonstrated a favorable profile of vaccine-induced T cell responses that might still provide a layer of COVID-19 protection.

**FUNDING:**

This study was funded by the National Centre for Infectious Diseases (NCID) Catalyst Grant (FY2021ES) and the National Research Fund Competitive Research Programme (NRF-CRP25-2020-0003).

## Graphical abstract

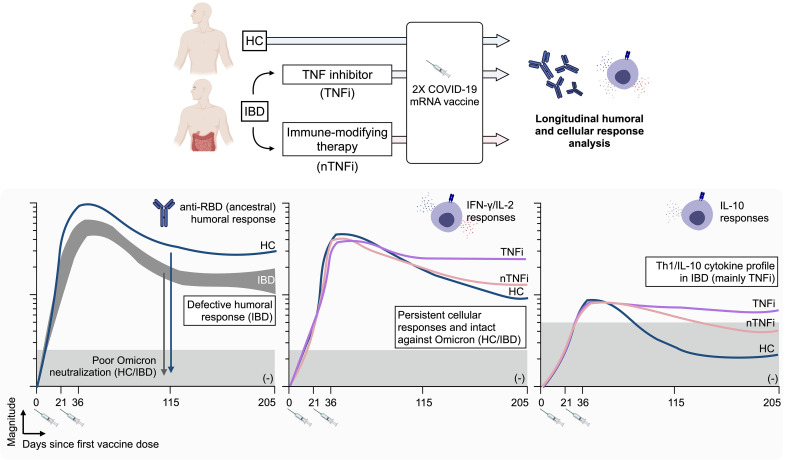

## Introduction

Immune-modifying agents are the treatment of choice for different chronic inflammatory diseases of autoimmune origin. Antimetabolites (azathioprine and methotrexate) or biologics, such as TNF inhibitors (adalimumab and infliximab), anti-p40 (ustekinumab), or anti-integrin (vedolizumab and etrolizumab) antibodies, are used alone or in combination to reduce inflammatory events in the gut (i.e., Crohn’s disease or ulcerative colitis), skin (i.e., psoriasis), joints (i.e., rheumatoid arthritis), or in multiple systems (i.e., systemic lupus erythematosus). While these agents reduce disease burden and improve quality of life (1), they are broadly considered immunosuppressive. The COVID-19 pandemic and the necessity of implementing widespread vaccination sparked debate and research on the effect of these chronic therapies on the immunogenicity of SARS-CoV-2 vaccination (2–4).

Others have already shown that these therapies, particularly TNF inhibitors, reduce the ability of different COVID-19 vaccines (based on mRNA or adenoviral vector) to produce spike-specific antibodies (5–8), especially those that recognize SARS-CoV-2 variants, including B.1.617.2 (Delta) (4). These results were expected, because reduced humoral responses to other vaccines (i.e., anti-pneumococcal, anti-HBV) have already been demonstrated in patients undergoing similar TNF inhibitor or other antimetabolite therapy (9–11) and because TNF-α has been demonstrated to play an important role in the coordination of humoral immunity maturation (12).

Nevertheless, antibodies can neither be considered the exclusive immunological parameter triggered by vaccination, nor the only determinant of its protective effect. Both mRNA- and adenoviral vector–based vaccines elicit humoral and cellular spike-specific immunity (13–15), and an early induction of spike-specific T cell responses is associated with the early protective effect of mRNA vaccination (16). In addition, the apparent indispensability of coordinated humoral and cellular immune activation for rapid and successful control of SARS-CoV-2 infection (17) and the rise to global circulation of the Omicron variant (18) both highlight the importance of the vaccine-induced T cell response. While spike mutations have conferred the Omicron variant with the ability to evade the majority of vaccine-induced neutralizing antibodies (19), spike-specific T cell immunity remains mainly intact against the Omicron variant (20–23).

While these T cells might not play a role in preventing infection, their ability to recognize and lyse virus-infected cells likely represents an important antiviral mechanism that might prevent the unchecked spread of SARS-CoV-2 in the infected host (24). However, the effect that different immune-modifying therapies exert on vaccine-induced spike-specific cellular immunity has only started to be analyzed (25), with initial evidence of preserved cellular immunity levels, at least immediately after vaccination.

In this paper, we therefore studied a cohort of patients with inflammatory bowel diseases (IBD) who are being treated with antimetabolites (AM), TNF inhibitor (TNFi), and/or other biologic treatments (anti-integrin and anti-p40, and we characterized both cellular and humoral vaccine-induced spike-specific immunity. Spike-specific immune responses were analyzed from before vaccination to 6 months following the second dose of COVID-19 mRNA vaccination (BNT162b2 or mRNA-1273). Importantly, we designed an experimental plan to investigate not only the ability of vaccine to elicit “classical” spike-specific T cell responses producing Th1 cytokines (IFN-γ and IL-2), but also the antiinflammatory/regulatory IL-10 cytokine. The rationale of such an experimental design was based on data demonstrating that TNFi therapy mediates induction of IL-10 in T cells that likely contribute to their ability to dampen inflammation (26, 27).

The ability to modify the functional profile of classical Th1 T cells can be of particular importance in SARS-CoV-2 infection. The presence and induction of both IL-10– and IFN-γ–producing SARS-CoV-2–specific T cells are associated with asymptomatic SARS-CoV-2 infection (28) and hybrid immunity (29), while their absence has been reported in severe COVID-19 (30). The induction of such T cells endowed with antiinflammatory potential might be advantageous in the asymptomatic control of SARS-CoV-2 infection. Furthermore, owing to the pervasion of the Omicron variant globally, the ability of vaccine-induced spike-specific T cells to tolerate the amino acid mutations that characterize the Omicron variant needs to be evaluated. We therefore directly tested ex vivo the effect that Omicron variant mutations exert on the spike-specific T cells induced in patients with IBD under different treatments.

## Results

### Study population.

**Figure 1:**
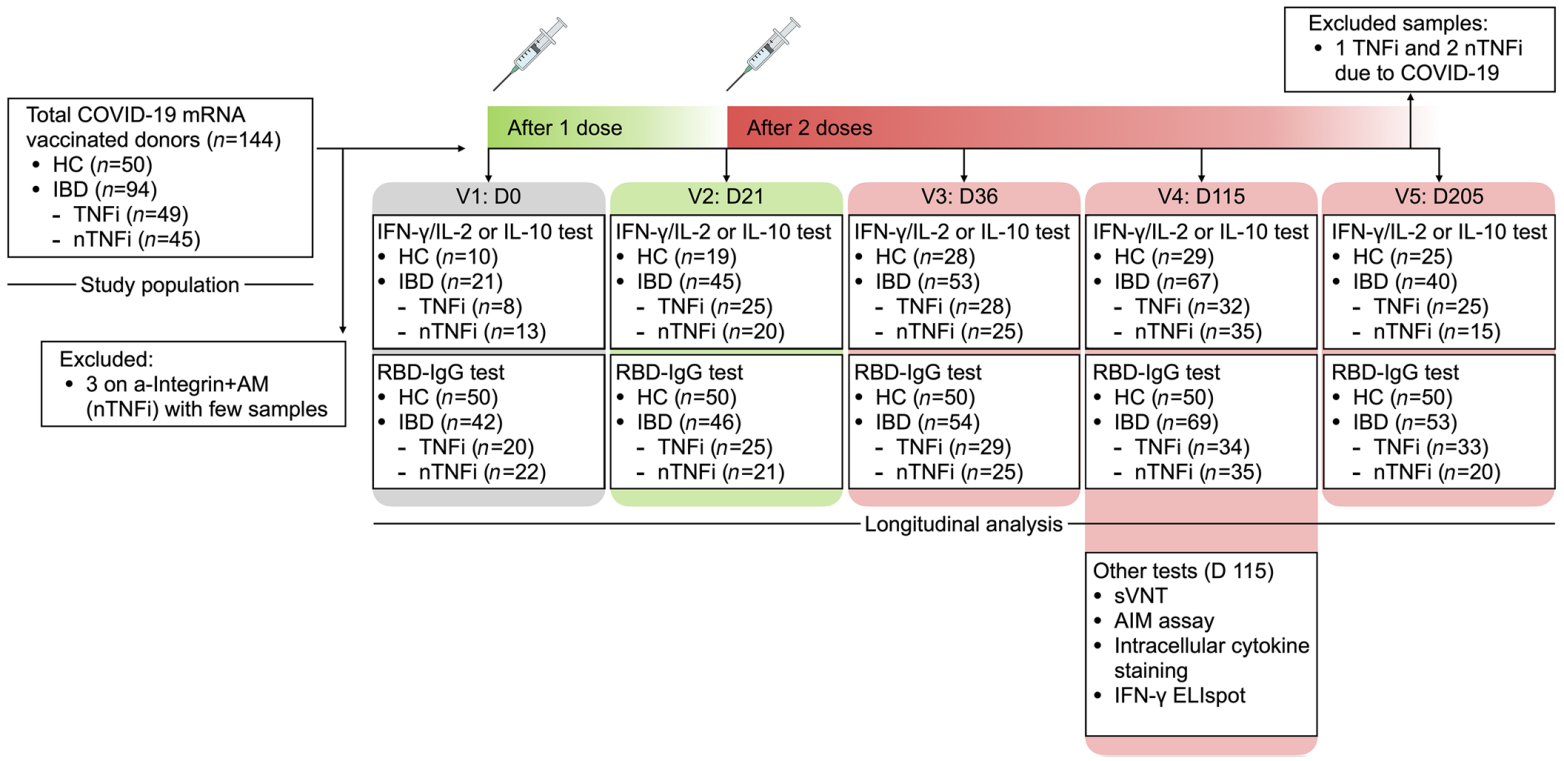
Study design outline. Peripheral blood samples from healthcare workers who were not on immune-modifying therapy and served as HCs or from patients with IBD on varying immunotherapies were collected for up to 5 study time points of interest. Humoral, cellular, and IL-10 responses were quantified longitudinally. Additional tests, including sVNT, AIM assay, intracellular cytokine staining, and IFN-γ ELIspot, were performed on a subset of donor samples obtained at the fourth study time point (day 115) for further analysis.

During the study period, 94 patients with IBD had at least 1 blood sample processed for analysis (Figure 1). Forty-five patients completed at least 3 visits, while the remaining patents were either lost to follow up or recruited after first or second vaccination. Of the 94 patients, 63 patients (67%) had Crohn’s disease and 31 patients (33%) had ulcerative colitis. Sixty-three patients (67%) with IBD and 18 healthy controls (HCs) (36%) were male, while 31 (33%) patients with IBD and 32 HCs (64%) were female (*P =* 0.0004). Median ages in IBD (41 yr) and HC groups (41 yr) were similar (*P =* 1). All HCs and 93 of the donors with IBD (99%) are of Asian ethnicity; 1 participant in the study is of White ancestry. Forty-nine patients (52%) were on TNFi, and 45 patients (48%) were on other non-TNFi (nTNFi) immunotherapy. Other baseline characteristics, including age, sex, IBD diagnosis and duration, mRNA vaccine taken, and steroid usage between TNFi and nTNFi groups, were similar (Table 1). Among those undergoing TNFi therapy, 27 (55%) had an additional antimetabolite. Among those on other nTNFi therapies were 15 patients (33%) only on an AM, 7 patients (16%) on anti-p40 (a-p40) monotherapy, and 11 patients (24%) on anti-integrin (a-Integrin) monotherapy. Those on an additional AM included 9 patients (20%) on a-p40 (a-p40+AM) and 3 patients (7%) on a-Integrin (a-Integrin+AM); those on a-Integrin+AM were excluded from analysis due to the lack of data points. Samples from 3 patients, all at the final time point (day 205), were collected but excluded from analysis because these patients were infected with SARS-CoV-2. Six patients were on concomitant steroid therapy: 2 in the TNFi group and 4 in the nTNFi group (*P =* 0.6). Eight patients from the TNFi group and 7 patients from the nTNFi group took the mRNA-1273 (Moderna) vaccine (*P =* 1). Further disease phenotype and behavior characteristics of the IBD cohort are shown in Table 2.

### Vaccine-induced humoral immunity.

**Figure 2:**
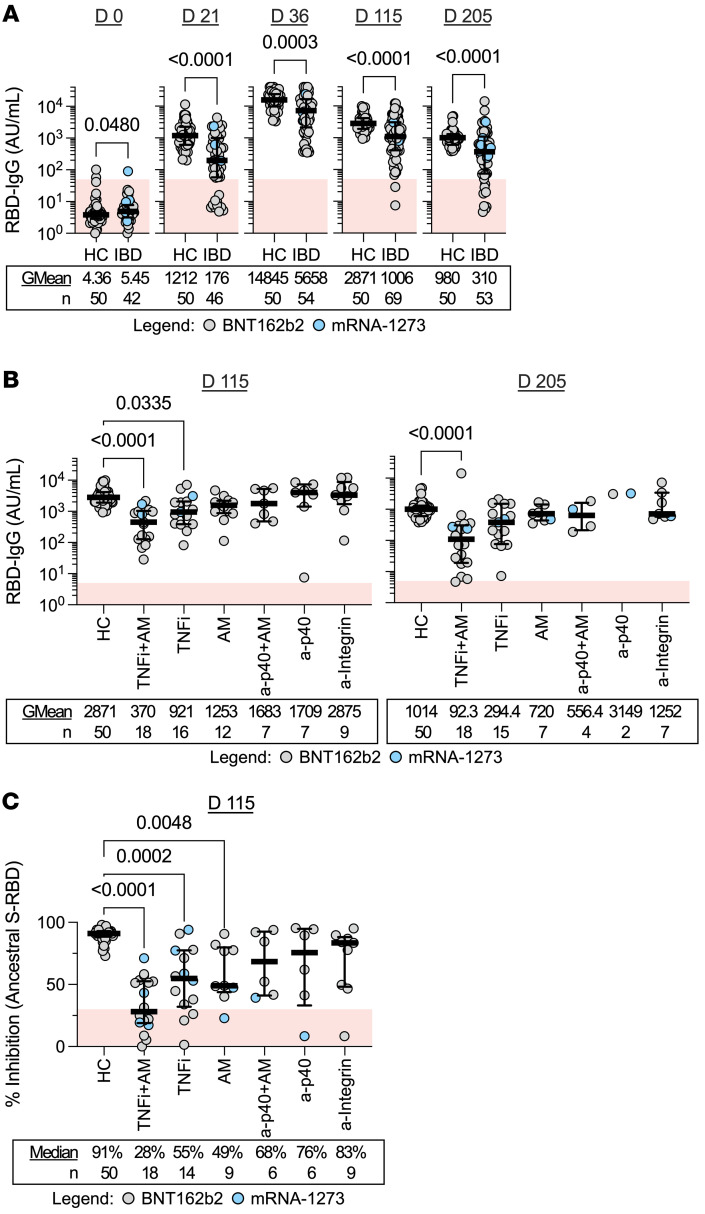
Humoral immunity is induced following COVID-19 mRNA vaccination. (**A**) Dot plots with median (middle bar) and interquartile range (whiskers) of RBD IgG concentrations (AU/mL) from serum samples of the 2 study cohorts collected at different time points. (**B**) RBD IgG concentrations (AU/mL) from serum samples of HCs and patients with IBD grouped by treatment 3 and 6 months after completing their 2-dose vaccination. (**C**) Percentage inhibition measured by sVNT of the ancestral SARS-CoV-2 S-RBD from serum samples of HCs and patients with IBD grouped by treatment 3 months after completing their 2-dose vaccination. For all graphs, the shaded red region denotes the area under the threshold for a positive test. Statistical analyses were performed by (**A**) Wilcoxon’s signed-rank test or by (**B** and **C**) Kruskal-Wallis and Dunn’s test, with *P* values indicated above the comparison line when significant (*P* ≤ 0.05). Geometric means (GMean; AU/mL) or median inhibition and number of data points (*n*) are indicated below each group.

SARS-CoV-2 spike receptor binding domain (RBD) IgG levels were quantified in response to COVID-19 mRNA vaccination both in the HCs and the IBD cohort (Figure 2). In line with previous observations (5–7), at all postvaccination time points — day 21 (just before second mRNA dose), day 36 (2 weeks after 2-dose vaccination), day 115 (3 months after 2-dose vaccination), and day 205 (6 months after 2-dose vaccination) — the medians of IBD cohort humoral responses (geometric means [GMean] of 176, 5658, 1006, and 310 AU/mL) were lower (*P <* 0.005) than what was observed in HCs (GMean of 1212, 14845, 2871, and 980 AU/mL; Figure 2A).

The deficiency of vaccine-induced anti-RBD antibodies was evident in patients undergoing therapy with TNFi monotherapy on day 115 and even more so in patients under combination therapy with TNFi and an antimetabolite (TNFi+AM; 3 and 6 months after 2-dose vaccination) (Figure 2B). No significant differences among anti-RBD titers 3 and 6 months after vaccination (days 115 and 205) in HCs and patients with IBD undergoing nTNFi therapies were observed. Patients treated with AM, a-p40, and a-Integrin therapies on day 115 and day 205 displayed anti-RBD titers that were indistinguishable from those of HCs. We also performed a surrogate viral neutralization test (sVNT) and found significantly reduced inhibition of ancestral S-RBD binding to human ACE2 (hACE2) in TNFi+AM, TNFi, and AM groups on day 115 (Figure 2C).

### Vaccine-induced spike-specific T cell responses.

**Figure 3:**
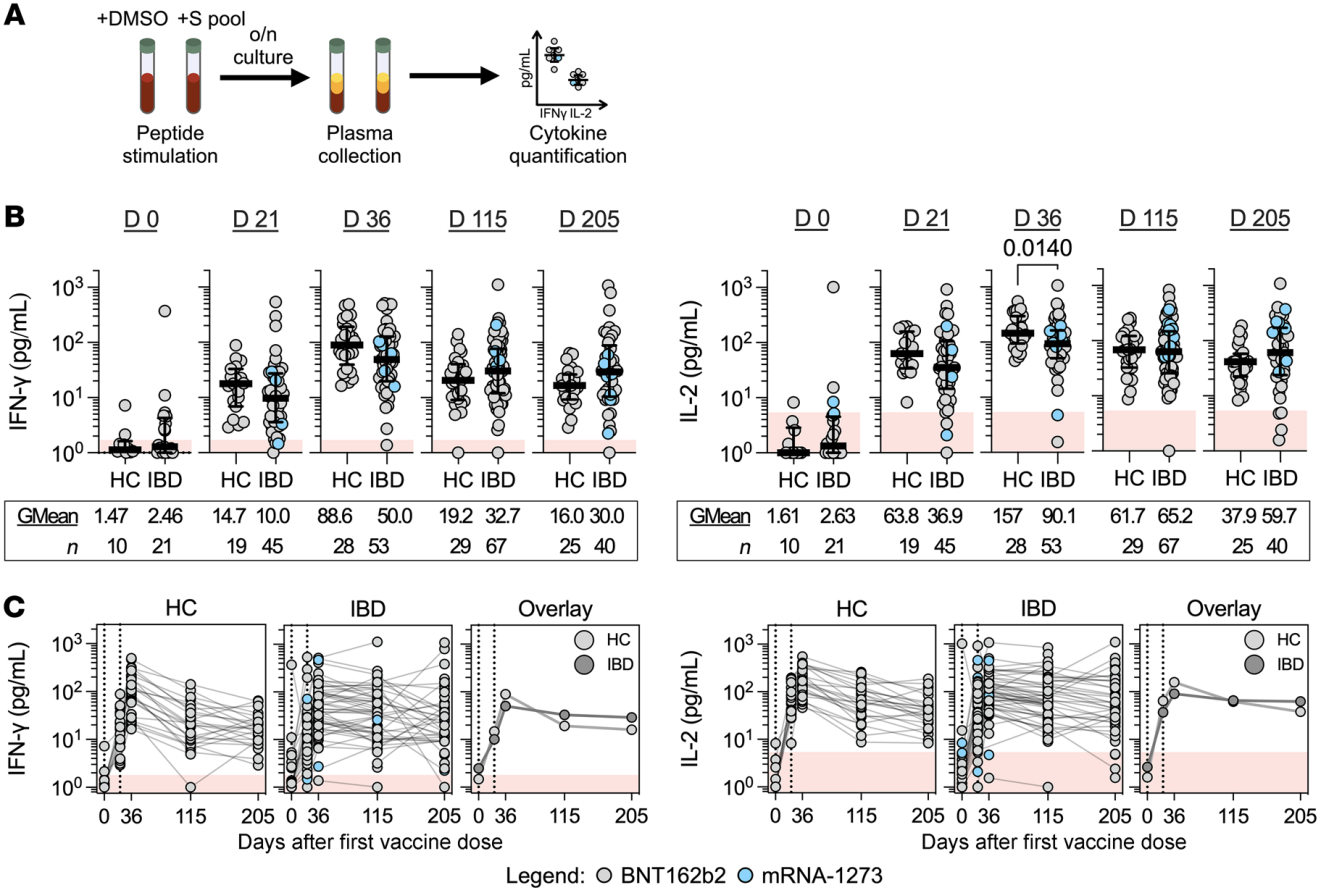
Cellular immunity is induced following COVID-19 mRNA vaccination. (**A**) Overview of whole blood cytokine release assay for IFN-γ/IL-2 quantification. (**B**) Dot plots with median (middle bar) and interquartile range (whiskers) of IFN-γ or IL-2 concentrations (pg/mL) from S pool–stimulated whole-blood supernatants of the 2 study cohorts collected at different time points. Statistical analyses were performed by Wilcoxon’s signed-rank test, with *P* values indicated above the comparison line when significant (*P* ≤ 0.05). Geometric means (GMean; AU/mL) and number of data points (*n*) are indicated below each group. (**C**) Quantified IFN-γ or IL-2 concentrations (pg/mL) plotted against time, faceted by the 2 study cohorts. Data points originating from the same participant are connected by gray lines. Data are summarized in the “Overlay,” plot with lines connecting the geometric means of each group at each sampling interval. For all graphs, shaded red regions denote the area under the threshold for a positive test.

The magnitude and function of the spike-specific T cell response induced by vaccination in HCs and patients with IBD was characterized directly in whole blood. A pool of 15-mer peptides covering the immunogenic regions of the SARS-CoV-2 S-protein (S pool) was used to measure spike-specific T cell responses (Supplemental Table 1). The quantity of Th1 cytokines (IFN-γ and IL-2) secreted in the plasma after peptide stimulation was quantified after overnight incubation (Figure 3A). This rapid quantitative assay is demonstrated to possess identical sensitivities/specificities of conventional ELISpot assays (31).

Before vaccination, whole-blood supernatants of HCs and patients with IBD stimulated with S pool displayed median IFN-γ and IL-2 levels below threshold. Some whole-blood supernatants from either cohort exhibited cytokine production higher than unstimulated controls, consistent with the presence of spike cross-reactive T cells already demonstrated in uninfected individuals (32, 33). Peptide-induced IFN-γ and/or IL-2 clearly increased in both HCs and patients with IBD after first- (day 21) and second-dose vaccination (day 36) in line with the ability of mRNA vaccines to induce spike-specific T cells (13). In particular, 2 weeks after the second vaccination, all HCs (28 of 28 for both IFN-γ and IL-2) and the majority of patients with IBD under immune-modifying therapies possessed positive IFN-γ (50 of 51) and IL-2 (49 of 51) responses (Figure 3B). Importantly, we did not observe any reduction of IFN-γ or IL-2 responses from 3 and 6 months after 2-dose vaccination in patients with IBD in comparison to HCs.

**Figure 4:**
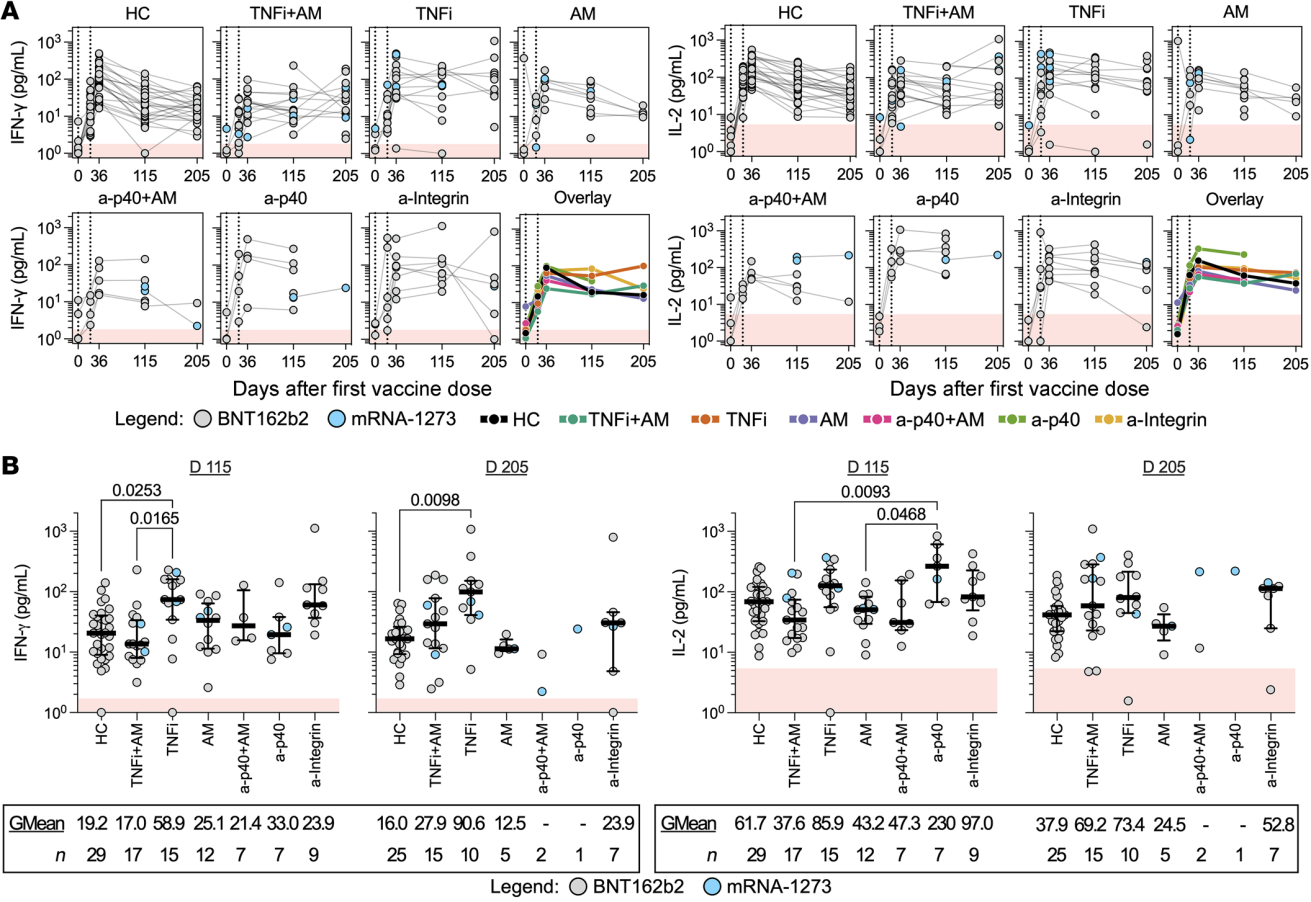
Durable T cell responses are demonstrated by patients under different immunotherapies. (**A**) Quantified IFN-γ or IL-2 concentrations (pg/mL) plotted against days after first vaccine dose for both HCs and patients with IBD grouped by treatment. Data points originating from the same participant are connected by gray lines. Data are summarized in the “Overlay,” plot with lines connecting the geometric means of each group at each sampling interval. (**B**) Dot plots with median (middle bar) and interquartile range (whiskers) of quantified IFN-γ or IL-2 concentrations (pg/mL) from S pool–stimulated whole-blood supernatants of HCs and patients with IBD grouped by treatment 3 and 6 months after completing their 2-dose vaccination. Statistical analyses were performed by Kruskal-Wallis and Dunn’s test, with *P* values shown above the comparison lines when significant (*P* ≤ 0.05). Geometric means (GMean; AU/mL) and number of data points (*n*) are indicated below each group. For all graphs, the shaded red region denotes the area under the threshold for a positive test.

We then analyzed cytokine levels induced in patients with IBD under different treatment regimens. Longitudinal responses from individual donors are displayed in Figure 4A. As shown in Figure 4B, patients treated with TNFi alone demonstrated higher IFN-γ response levels than HCs at both 3 and 6 months after 2-dose vaccination. Although few, we also noted that patients treated with a-p40 biologics present high median levels of IL-2 production 3 months after 2-dose vaccination (*n =* 7, GMean 229.6 pg/mL). Importantly, no specific treatment caused an inhibition of the quantity of IFN-γ and IL-2 (days 115 and 205) in comparison with HCs; even the TNFi+AM group displaying lower spike-specific humoral responses (Figure 2) and lower induction of T cell responses on day 36 (Supplemental Figure 1) demonstrated IFN-γ and IL-2 responses comparable to those in HCs at these later time points.

We also compared the magnitude of spike-specific T cell responses between patients with IBD who were vaccinated with either BNT162b2 or mRNA-1273. No differences were observed in IFN-γ and IL-2 quantities at all postvaccination time points, aside from an increased production of IL-2 in mRNA-1273–vaccinated patients with IBD on day 115 and day 205 (Supplemental Figure 2A). The finding of higher levels of IFN-γ responses in TNFi-treated patients with IBD 3 and 6 months after 2-dose vaccination was maintained when mRNA-1273–vaccinated donors were excluded from the analysis (Supplemental Figure 2, B and C).

Thus, mRNA vaccination in patients with IBD undergoing treatment with different immune-modifying therapies demonstrated a spike-specific T cell cytokine responses that was not inferior to what was detectable in HCs. Furthermore, TNFi therapy was associated with a level of spike-specific T cell responses 3 and 6 months after second vaccination (days 115 and 205) greater than that of HCs.

### Spike-specific CD4^+^ and CD8^+^ T cells in vaccinated patients with IBD and impact of Omicron variant mutations.

**Figure 5:**
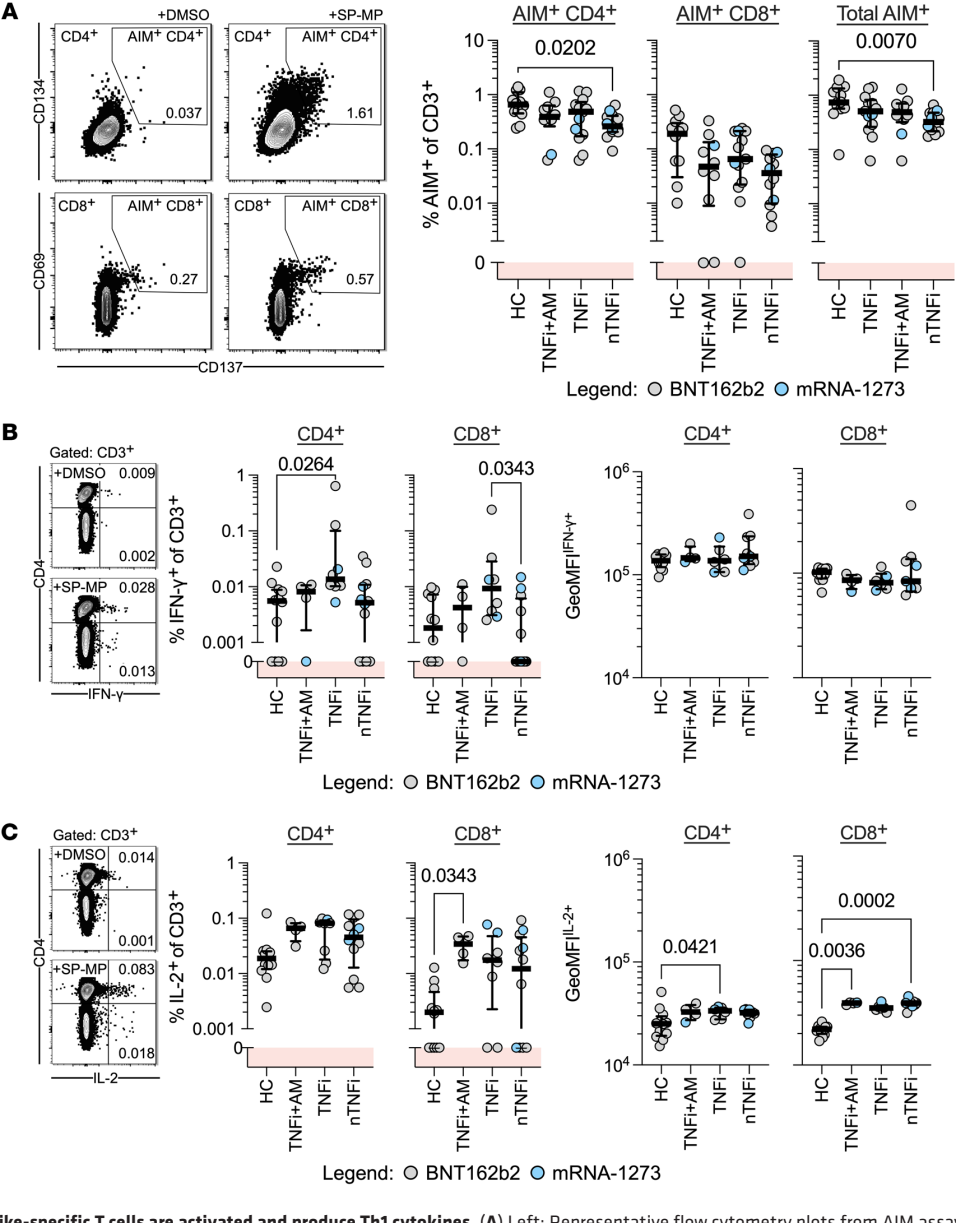
Spike-specific T cells are activated and produce Th1 cytokines. (**A**) Left: Representative flow cytometry plots from AIM assays identify CD4^+^CD134^+^CD137^+^ and CD8^+^CD69^+^CD137^+^ T cell populations with or without stimulation with spike peptides. Activated cells are defined by the drawn gate within each population. Right: Summary frequencies of AIM^+^ cells identified in PBMCs from HCs or patients with IBD 3 months after the second vaccine dose (HC, *n =* 12; TNFi+AM, *n =* 10; TNFi, *n =* 13; nTNFi, *n =* 12). (**B** and **C**) Intracellular cytokine staining for (**B**) IFN-γ^+^ or (**C**) IL-2^+^ T cell populations with or without stimulation with spike peptides. Representative flow cytometry plots and dot plots (with median and IQR) summaries of CD4^+^ or CD8^+^IFN-γ^+^ or IL-2^+^ frequencies of CD3^+^ cells and background-subtracted geometric mean fluorescence intensities (GeoMFIs) of IFN-γ^+^ or IL-2^+^ populations (HC, *n =* 12; TNFi, *n =* 8; TNFi+AM, *n =* 4; nTNFi, *n =* 12). Statistical analyses were performed by Kruskal-Wallis and Dunn’s test, with *P* values shown above the comparison lines when significant (*P* ≤ 0.05). For all graphs, the shaded red regions denote responses below background levels (denoted with 0).

To confirm that COVID-19 mRNA vaccination induces spike-specific CD4^+^ and CD8^+^ T cells in patients with IBD undergoing immune-modifying therapy, PBMCs collected on day 115 from patients with IBD were stimulated with a spike peptide megapool (SP-MP) and analyzed for expression of activation-induced markers (AIMs) on gated CD4^+^ and CD8^+^ T cells (Figure 5A). Peptide stimulation activated more CD4^+^ T cells than CD8^+^ T cells in all donor groups. Furthermore, while a lower frequency of AIM^+^CD4^+^ T cells was found in nTNFi donors than in HCs, TNFi/TNFi+AM patients presented frequencies of AIM^+^CD4^+^ and CD8^+^ T cells similar to those of HCs.

The higher quantity of IFN-γ detected in peptide-stimulated whole blood of donors with IBD on TNFi therapy could either be related to a cumulative increase in the cytokine secretion potential of individual spike-specific T cells or a larger fraction capable of IFN-γ secretion, rather than an increase in their frequency. Therefore, we quantified spike specific CD4^+^ and CD8^+^ T cells able to produce IFN-γ and IL-2. Although low frequencies were detected, CD4^+^IFN-γ^+^ T cells in TNFi-treated donors with IBD were enriched relative to those in HCs, while geometric MFIs (GeoMFI) of either CD4^+^ or CD8^+^ IFN-γ among TNFi-treated donors and HCs were similar (Figure 5B). Interestingly, levels of IL-2^+^CD4^+^ or CD8^+^ spike-specific T cells were marginally higher in patients with IBD than in HCs, reaching statistical significance for CD8^+^ T cells in TNFi+AM-treated patients. Moreover, IL-2 GeoMFIs were significantly elevated in the spike-specific CD4^+^ T cells of TNFi-treated donors and in CD8^+^ T cells of TNFi+AM and nTNFi-treated donors than in HCs (Figure 5C). These findings hint that, while vaccination under concurrent TNF inhibition induced similar amounts of antigen-specific T cells, HCs and TNFi-treated patients with IBD differed in IFN-γ– and IL-2–producing fractions.

**Figure 6:**
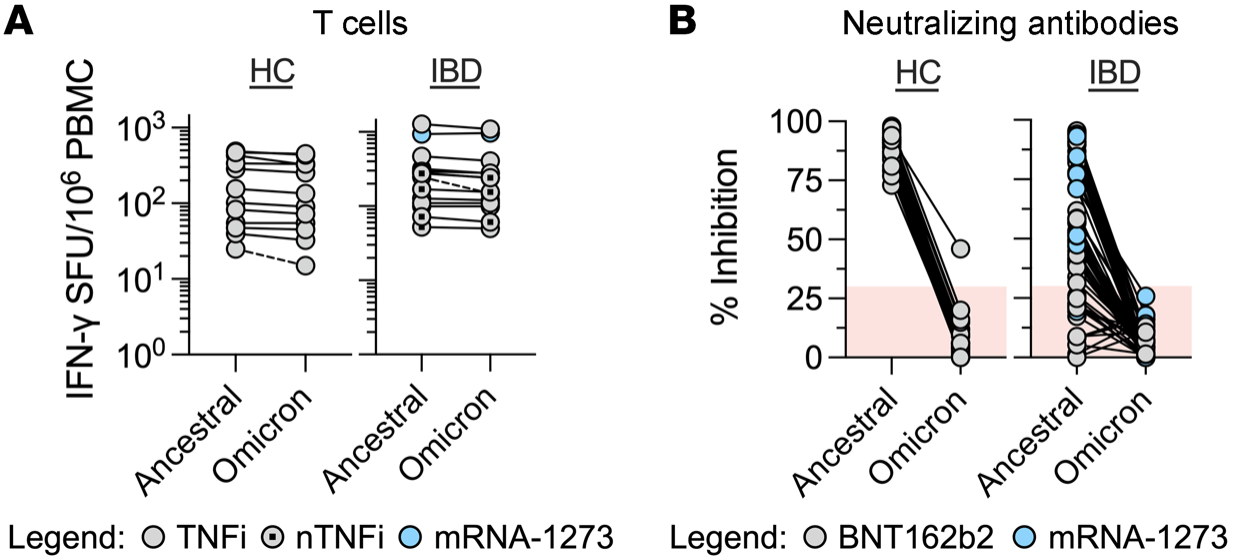
Cellular but not humoral responses are mostly preserved against Omicron variant spike. (**A**) Dot plots denoting the number of IFN-γ SFU per 10^6^ PBMCs generated after ancestral and Omicron variant spike peptide stimulation in HCs or IBD donor groups (day 115). Each line connects paired responses from a single donor, with broken lines denoting a difference of more than 25% responses (HC, *n =* 12; TNFi or TNFi+AM [TNFi ± AM], *n =* 8; nTNFi, *n =* 6). (**B**) Dot plots denoting the percentage inhibition of binding of SARS-CoV-2 S-RBD to hACE2 by sVNT from donor sera (day 115). Each line connects paired responses from a single donor (HC, *n =* 50; IBD, *n =* 63). Shaded red regions denote data with negative result calls.

We then analyzed the effect of the mutations that characterize the spike protein of the Omicron variant on the vaccine-induced spike-specific T cells present in HCs and in patients with IBD (Figure 6). Patient PBMCs were stimulated with 3 peptide pools covering the entire spike protein (253 peptides) of the ancestral SARS-CoV-2 (Supplemental Table 2) and the regions mutated in the Omicron variant (67 peptides), with and without the amino acid substitutions/deletions that characterize the SARS-CoV-2 Omicron variant (Supplemental Table 3). We performed an IFN-γ ELISpot assay to quantify the frequency of SARS-CoV-2–specific T cells responding to conserved regions of the spike protein and to derive the frequency of responses altered by the variant-defining regions in the Omicron variant. As already seen in healthy vaccinated individuals (20–23), the spike-specific T cell response to the Omicron variant was mainly preserved in all HCs and donors with IBD irrespective of their treatment. An inhibition of more than 25% of the total spike-specific T cell response due to Omicron mutations was observed in only 1 of 12 HC and 1 of 14 patient with IBD samples tested (Figure 6A). In contrast, pairwise analysis of neutralizing antibodies in day 115 donor sera by sVNT of both the ancestral and Omicron variant S-RBD demonstrated a dramatic decrease to below-threshold levels (<30% inhibition) in virtually all tested samples (49 of 50 HCs and 63 of 63 patients with IBD) for hACE2-RBD binding inhibition (Figure 6B).

### Immune-modifying therapies increase IL-10 production of spike-specific T cells.

**Figure 7:**
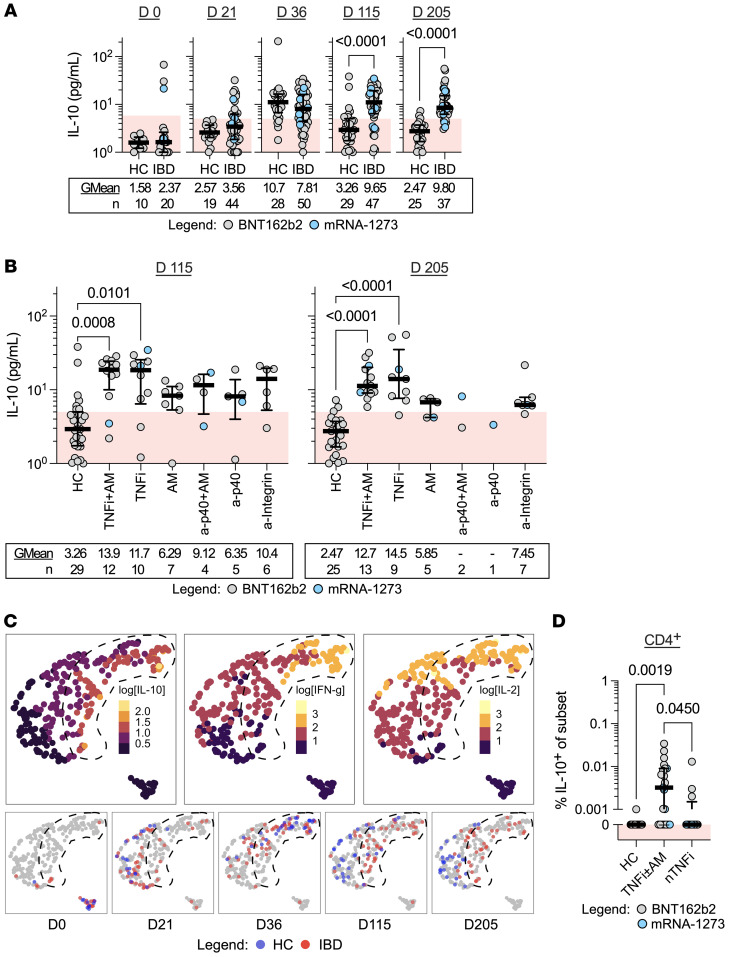
IL-10 delineates T cell cytokine response profile of individuals undergoing immune-modifying therapies. (**A** and **B**) Dot plots with median (middle bar) and interquartile range (whiskers) (**A**) of IL-10 concentrations (pg/mL) from S pool–stimulated whole-blood supernatants of the 2 study cohorts collected at different time points and (**B**) of HCs and patients with IBD grouped by treatment 3 and 6 months after completing their 2-dose vaccination. Shaded red regions denote the area under the threshold for a positive test. Statistical analyses were performed by (**A**) Wilcoxon’s signed-rank test or (**B**) Kruskal-Wallis and Dunn’s test, with *P* values indicated above the comparison line when significant (*P* ≤ 0.05). Geometric means (GMean; AU/mL) and number of data points (*n*) are indicated below each group. (**C**) UMAP projections based on IL-10, IFN-γ, and IL-2 quantities measured from each donor time point. Images on the top row display each point filled according to log_10_-transformed cytokine quantities (pg/mL). Images on the bottom row display points filled according to study cohort at the respective time point. A shape is drawn enclosing a region mostly containing points with IL-10 values of greater than 10 pg/mL. (**D**) Dot plots with median (middle bar) and interquartile range (whiskers) of IL-10^+^CD4^+^ cell frequencies of CD4^+^ from HCs (*n =* 12) or donors with IBD on TNFi (with or without AM, *n =* 21) or nTNFi therapy (*n =* 12). Statistical analysis was performed by Wilcoxon’s signed-rank test, with *P* values indicated above the comparison line when significant (*P* ≤ 0.05). For all graphs, the shaded red region denotes responses below background levels (denoted with 0).

Differences in the kinetics of spike peptide–induced IFN-γ and IL-2 detected in patients with IBD undergoing TNFi therapy suggest that this treatment might modify vaccine-induced spike-specific T cells. In addition, TNFi therapy has been shown to modify T cell function through expression of a transcriptional signature that upregulates IL-10 production in T cells (27). We therefore tested whether cytokine secretion profiles in whole-blood supernatants after spike-peptide stimulation contain not only classical Th1 cytokines IFN-γ and IL-2, but also IL-10. The quantity of IL-10 detected in treated patients with IBD and HCs before and after 2-dose vaccination was measured (Figure 7).

At time points following first- (day 21) and second-dose vaccination (day 36), increased concentrations of IL-10 were detected in whole-blood supernatants of HCs and patients with IBD relative to their respective prevaccination baselines. Furthermore, at 3 and 6 months after the second vaccine dose (day 115 and day 205), no IL-10 was detected in the majority of HCs, while we noticed a persistence of IL-10 induction in patients with IBD (Figure 7A). Values of quantified IL-10 (e.g., 9.65 pg/mL on day 115) were low in comparison to corresponding IL-2 (61.5 pg/mL) and IFN-γ (31.5 pg/mL) responses in patients with IBD. Particularly, sustained production of IL-10 in peptide-stimulated whole blood was observed in both TNFi-treated subcohorts both at 3 and 6 months after 2-dose vaccination (Figure 7B).

To characterize the chronological evolution of cytokine profiles, we used UMAP to integrate quantified, log-transformed IL-10 data with IFN-γ and IL-2 for each donor time point (Figure 7C). UMAP projections of data points originating from prevaccination samples of either HCs or patients with IBD formed a distinct cluster. Moreover, the data points cosegregated following the first dose (day 21) and 2 weeks after second-dose vaccination (day 36), further highlighting the similarities of cellular responses between the 2 cohorts. Notably, 3 months after completion of the 2-dose regimen (day 115), the cytokine profiles diverged into distinct clusters, with profiles of patients with IBD coinciding with regions defined by higher levels of IL-10, IFN-γ, and IL-2. This observed clustering persisted up to 6 months after 2-dose vaccination. Further analysis showed that IL-10 production did not correlate with either IFN-γ or IL-2 in HCs, while a significant but weak correlation existed between IFN-γ and IL-10 2 weeks (day 36) and 3 months (day 115) after the second dose in patients with IBD (Supplemental Figure 3).

To then confirm that spike peptide pool stimulation induces IL-10 production in T cells, we performed direct ex vivo intracellular staining of donor PBMCs on 3-month (day 115) samples. Indeed, the low magnitude of cumulative IL-10 that we observed in whole-blood-stimulated supernatants implied the identification of IL-10^+^ T cells to be a technically challenging feat. Intracellular cytokine staining revealed IL-10 accumulation upon spike peptide pool stimulation mainly detected in CD4^+^ T cells (Supplemental Figure 4, A and B). Furthermore, only IL-10^+^CD4^+^ T cells were enriched in PBMCs of patients with IBD on TNFi/TNFi+AM (TNFi±AM) (Figure 7D and Supplemental Figure 4C). None of the samples demonstrated distinct populations of T cells costaining for both IFN-γ and IL-10 intracellularly (Supplemental Figure 4, D and E), suggesting that production of these cytokines may occur independently or that the method utilized is unsuitable for costaining analysis.

## Discussion

The attenuated humoral responses detected in SARS-CoV-2–vaccinated patients under different immune-modifying treatments, particularly in those treated with TNFi therapy, have generally been interpreted to imply reduced vaccine immunogenicity, fueling debate on the possible increased risk of severe COVID-19 in patients treated chronically with these immune-modifying therapies (2, 3, 6, 7). Here, by studying patients with IBD under various regimens and vaccinated with the prevailing spike-based mRNA vaccines, we demonstrated that a spike-specific T cell response is not only induced in IBD-treated patients to levels similar to those in HCs, but that they also persisted longer and at higher levels, particularly in those patients treated with TNFi.

In contrast to antibodies, T cells cannot prevent infection; instead, they excel in the clearance of intracellular pathogens either through recognition and lysis of infected cells or through activating macrophages and supporting B cell maturation (24). Furthermore, because coordination between humoral and cellular arms of immunity is likely to be essential for rapid viral control and reduced pathogenicity (17), we cannot claim that the increased T cell immunogenicity observed directly translates into better protective efficacy of vaccination in patients under immune-modifying therapies. Nevertheless, these patients, particularly those undergoing TNFi therapy, were clearly able to mount a robust spike-specific cellular immunity. Additionally, TNFi therapy did not abolish but only reduced production of antibodies after mRNA vaccination. Previous observations (5–8) and our own data demonstrate this. It is possible therefore to hypothesize that the presence of cellular immunity against spike compensates for the observed humoral defect.

In this aspect, the demonstration provided here that vaccine-induced spike-specific T cells of patients with IBD are minimally altered in their ability to recognize Omicron variant spike adds a further layer of reassurance. Several recent works have shown that vaccine-induced spike-specific T cells in healthy individuals are mainly preserved against the Omicron variant (20–23). Our data in patients with IBD under different immune-modifying treatments demonstrated a similar pattern of reduced recognition only in a minority of tested patient samples. This finding suggests that, as in healthy vaccinated individuals (20–23), the spike-specific T cells of patients undergoing various immune-modifying regimens mount a multispecific T cell response against different conserved regions of spike. Therefore, vaccinated patients undergoing TNFi therapy may develop reliable protection against severe disease. By analyzing the kinetics of the spike-specific T cell response, we observed that the higher levels of IFN-γ secretion present 3 and 6 months after vaccination in TNFi-treated patients, in comparison to HCs, did not derive from a higher level of vaccine-induced spike-specific T cell induction at earlier time points; rather, they more likely derived from a propensity of the T cell response to persist longer. Our findings can be explained by the differential effect of TNF-α on humoral and cellular immune responses. While TNF-α downregulates T cell expansion (34), it supports B cell maturation (12). Therefore, in the context of vaccination, TNF-α inhibition can cause a reduction of subsequent B cell maturation with reduced antibody quantities (12) but a persistence of vaccine-induced T cells (34).

The inhibition of TNF-α, directly through TNFi or indirectly through other immunomodulatory treatments, can also explain the simultaneous induction of IFN-γ, IL-2, and IL-10 found in patients with IBD under different treatments. Blockade of the effect of TNF-α on T cells with TNF-α inhibitors is known to upregulate IL-10 in T cells (27). The presence of spike-specific Th1- and IL-10–producing cells can be advantageous in SARS-CoV-2 infection. Animal models have shown that the ability of T cells to secrete IFN-γ and IL-10 simultaneously led to effective viral control without triggering severe pathological processes (35–37). Previously, we also observed that a pattern of cytokine production, characterized by the simultaneous presence of IFN-γ, IL-2, and IL-10, constitutes the T cell response detected in patients who control SARS-CoV-2 infection asymptomatically (28). The importance of IL-10 and IFN-γ production by T cells has also been highlighted by two recent works: such a functional T cell profile was demonstrated to be defective in severe COVID-19 (30), while the presence of IL-10–producing spike-specific T cells is characteristic of individuals with hybrid SARS-CoV-2 immunity (29) who demonstrate a robust immunity from reinfection (38, 39). Regardless of our inability to visualize T cells coexpressing IL-10 and IFN-γ by ICS, the aggregate production of IL-10 and IFN-γ, particularly in TNFi-treated donors, may deliver functionally similar outcomes. Of note, the demonstration that mRNA vaccination in patients with IBD undergoing TNF inhibition resulted in the induction of T cells with an IFN-γ/IL-2/IL-10 secretion profile suggests that similar functional profiles might likewise be induced in virus-specific T cells after SARS-CoV-2 infection, explaining the clinical observation that SARS-CoV-2 infection in patients undergoing TNFi treatment is generally mild (40–42). In our own study, 6 patients, 4 of whom were on TNFi, who eventually developed COVID-19 all had a mild disease course and did not require hospitalization.

There are some limitations to this study — the most important of these being that the bulk of T cell experiments were performed not by direct measurement of T cell quantity, but by measuring cytokines secreted in whole blood after specific peptide stimulation. However, we provided direct evidence orthogonally by demonstrating spike-specific CD4^+^ and CD8^+^ T cells induced by vaccination in patients with IBD and visualizing IL-10^+^, IFN-γ^+^, and IL-2^+^ T cells upon spike peptide stimulation. Nevertheless, while whole blood cytokine release does not directly quantify the number of antigen-specific T cells, it provides a standardized method well suited for longitudinal analysis of T cell responses in patients under different treatments. The simplicity of the assay reduces the interassay variability and is directly performed on fresh whole blood, limiting the detrimental effects of freezing and thawing (43). Furthermore, because T cell functionality is analyzed in whole blood, the immune-modifying therapies administered into the patients are present at therapeutic levels during the assay, mimicking, as we previously argued (44), more closely the situation in vivo.

In conclusion, we have shown here that mRNA vaccination in patients with IBD under different immunomodulatory treatments triggered a robust cellular immune response amidst an attenuated humoral response. Particularly, patients under TNFi monotherapy demonstrated reduced kinetics of decline of spike-specific T cell responses and an ability to secrete a cytokine profile characterized by the simultaneous presence of IFN-γ, IL-2, and the antiinflammatory IL-10 cytokine upon spike encounter. Since this T cell functional profile has been preferentially associated with asymptomatic SARS-CoV-2 control (28), COVID-19 mRNA vaccination in individuals under such immunomodulatory therapies might still offer a layer of protection. Moreover, these may even offer some advantages in controlling SARS-CoV-2 infection with limited pathological sequelae.

## Methods

### Study design.

This is a prospective, observational study conducted to assess both humoral and cellular responses to mRNA-based COVID-19 vaccines (BNT162b2 and mRNA-1273) in patients with IBD who were treated with antimetabolites, TNFi, and/or other biologics from July 2021 to February 2022. Specifically, the included therapies were azathioprine or methotrexate for antimetabolites, adalimumab or infliximab for TNFi, ustekinumab for anti-p40, and vedolizumab or etrolizumab for anti-integrin. Patients had completed 2 same-dose vaccine courses 3 weeks apart with either one of the COVID-19 mRNA vaccines (*n =* 94). The HC group included healthcare professionals not undergoing immune-modifying therapy (*n =* 50). Patients younger than 18 years old, those with previous SARS-CoV-2 polymerase chain reaction–confirmed COVID-19, or pregnant women were all excluded. Demographic data were self-reported based on national registry classification. Samples were collected at baseline before vaccination (day 0), 3 weeks (day 21 ± 5 days) after first dose of vaccine, 2 weeks (day 36 ± 5 days) after second dose of vaccine, 3 months (day 115 ± 5 days) after second dose of vaccine, and 6 months (day 205 ± 5 days) after second dose of vaccine. Due to the rapidity of vaccination uptake, recruitment of patients before vaccination became more challenging. Hence, the protocol was extended to include longitudinal follow-up of patients with IBD who were on antimetabolites/biologics and received their first and/or second dose of vaccine according to the study interval for blood sampling.

### Quantification of humoral responses.

Measurements were performed using the Abbott Architect i2000 automated analyzer using the SARS-CoV-2 IgG II Quant assay (Abbott), a chemiluminescent microparticle immunoassay for the quantitative detection of IgG targeting the RBD of the S1 subunit of the spike protein of SARS-CoV-2. Results are expressed as AU/mL, where values equal to or more than 50.0 AU/mL were interpreted as positive.

### Surrogate virus neutralization test.

Inhibition rates for hACE2 binding to S-RBD by neutralizing antibodies were derived using the GenScript SARS-CoV-2 Surrogate Virus Neutralization Test kit according to the manufacturer’s protocol and equation, with the ancestral and Omicron variant SARS-CoV-2 S-RBD. Patient sera were diluted 1:19 with sample dilution buffer and combined 1:1 with either ancestral SARS-CoV-2 HRP-RBD or Omicron variant HRP-RBD for 30 minutes at 37°C. The resulting mixture was added onto the hACE2 receptor–coated capture plate and incubated for 15 minutes at 37°C. The plate was washed 4 times before incubation in TMB solution. The reaction was stopped with Stop Solution (GenScript), and the solutions were read at 450 nm in a microtiter plate reader (Tecan Spark 10M). A negative result was called for values of less than 30% signal inhibition.

### Quantification of cellular responses and analysis.

We used a cytokine release assay (CRA) of whole peripheral blood stimulated using a SARS-CoV-2 spike–derived peptide S pool (Supplemental Table 1) described previously (31). Freshly drawn whole blood (320 μL; within 6 hours of venipuncture) was mixed with 80 μL RPMI and stimulated with S pool peptides to a final peptide concentration of 2 μg/mL or mixed with an equivalent amount of DMSO as control. Culture supernatants were collected 16 hours after culture and stored at −80°C until cytokine quantification. IFN-γ/IL-2 or IL-10 concentrations in plasma were quantified using an Ella machine (ProteinSimple) with microfluidic multiplex cartridges following the manufacturer’s instructions. Background cytokine levels quantified from DMSO controls were subtracted from the corresponding peptide pool–stimulated samples. The threshold for a positive response was set at 10 times the lower limit of quantification for each cytokine (IFN-γ = 1.7 pg/mL; IL-2 = 5.4 pg/mL; IL-10 = 5.8 pg/mL). A pseudocount of 1 pg/mL was applied to the data set for logistic transformation. Subsequently, log-transformed concentrations of each cytokine in all culture supernatants were projected onto UMAP space using 15 nearest neighbors, min_dist of 0.2 and Euclidean distance.

### PBMC separation.

PBMCs from HBSS-diluted anticoagulated blood (1:1) were separated by Ficoll-Paque density gradient centrifugation. PBMCs were frozen in FBS containing 10% DMSO and stored in liquid nitrogen until use.

### ELISpot assay.

ELISpot plates (Millipore) were coated with human IFN-γ antibody overnight at 4°C. Cryopreserved PBMCs were thawed and seeded at a density of 400,000 cells per well and stimulated with a respective peptide pool for 18 hours (2 μg/mL) or an equivalent amount of DMSO (negative control). The plates were then incubated with human biotinylated IFN-γ detection antibody (Mabtech), followed by Streptavidin-AP (Mabtech), and developed using the KPL BCIP/NBT Phosphatase Substrate (SeraCare). To quantify positive peptide-specific responses, twice the number of mean spots of the unstimulated wells was subtracted from the peptide-stimulated wells, and the results are expressed as spot-forming cells (SFU)/10^6^ PBMCs. Results were excluded if negative control wells had more than 30 SFU/10^6^ PBMCs or positive control wells (PMA/ionomycin) were negative.

### Measurement of the effect of the Omicron variant on total spike-specific T cells.

We directly tested donor PBMCs by IFN-γ ELISpot for reactivity against the ancestral or Omicron variant spike protein. To quantify total responses to the ancestral spike, we used a 10–amino acid overlapping 15-mer peptide pool (SP-MP) covering the entire spike protein listed in Supplemental Table 2 (1273 amino acids). For Omicron variant spike responses, we designed 2 peptide pools (Supplemental Table 3): one consisting of ancestral-derived spike peptides covering the variable regions (termed the “spike Hotspot-Ancestral” pool) and another consisting of the Omicron-derived spike peptides covering the same region (termed the “spike Hotspot-Omicron” pool). From this, we derived the total SFUs formed against the entire Omicron variant spike using the following equation: SFU_total_ _Omicron_ _spike_ = SFU_SP-MP_ – SFU_spike_ _Hotspot-Ancestral_ + SFU_spike_ _Hotspot-Omicron_. From this, the percentage of inhibition due to variation in Omicron variant spike sequences may be quantified using the following equation: Inhibition = SFU_SP-MP_ – SFU_total Omicron spike_/SFU_SP-MP._

### AIM assay.

For each condition, 1 million PBMCs in 150 μL AIM-V + 2% AB were stimulated for 24 hours at 37°C with a megapool (2 μg/mL) of 15-mer peptides encompassing the full spike protein (SP-MP) or an equivalent amount of DMSO in the presence of 1 μg/mL CD28/CD49d costimulation (BD). Cells were then washed in FACS buffer (1X PBS, 1% BSA, and 0.1% sodium azide) with 2 mM EDTA and stained with surface markers (CD3, CD4, CD8, CD69, CD134 and CD137 mAbs) diluted in FACS buffer (room temperature for 25 minutes). Dead cells were excluded using the Fixable Yellow Live/Dead fixable cell stain kit (Invitrogen). After 2 more washes in FACS buffer, the cells were resuspended in PBS + 1% FA prior to analysis. The gating strategy is outlined in Supplemental Figure 5, and the staining reagents used are outlined in Supplemental Table 4. Reported frequencies of AIM^+^ cells are background subtracted from DMSO/unstimulated samples, with a pseudocount of 10^-4^ added to represent below-background or null (zero) frequencies in log-scale.

### Intracellular cytokine staining.

For each condition, 1 million PBMCs in 150 μL AIM-V + 2% AB were stimulated for 24 hours at 37°C with SP-MP (2 μg/mL) or an equivalent amount of DMSO in the presence of 1 μg/mL CD28/CD49d costimulation (BD). In the last 4 hours, 1 μg/mL Brefeldin A and 0.5X Monensin (Biolegend) were added. Cells were then washed in FACS buffer containing 2 mM EDTA and stained with surface markers (CD3, CD4, and CD8 mAbs) diluted in FACS buffer (room temperature for 25 minutes). Dead cells were excluded using the Fixable Yellow Live/Dead fixable cell stain kit (Invitrogen). Cells were washed twice in FACS buffer and fixed in Cytofix/Cytoperm (BD) for 20 minutes on ice. Cells were then washed with Perm/Wash (BD) solution prior to intracellular staining (anti-IFN-γ, anti-IL-2, or anti-IL-10). After 2 more washes in FACS buffer, the cells were resuspended in PBS + 1% FA prior to analysis. Similar to above, the gating strategy is outlined in Supplemental Figure 5, and the staining reagents used are outlined in Supplemental Table 4. Reported frequencies of cells staining for cytokines are background-subtracted from DMSO/unstimulated samples, with a pseudocount of 10^–5^ added to represent below-background (zero) or null frequencies in log-scale. GeoMFIs reported for positive-staining populations are subtracted from negative-staining population GeoMFIs.

### Flow cytometry.

All flow cytometry samples were analyzed using cryopreserved cells that were thawed and resuspended in AIM-V media supplemented with 2% AB serum. Samples were stained accordingly and fixed in PBS + 1% FA. Acquisition was performed on a BD-LSR II Analyzer (BD) or CytoFLEX S (Beckman Coulter) within 24 hours and analyzed with FlowJo (BD)

### Statistics.

Statistical analyses were performed using R Statistical Software (version 4.0.3) (ggpubr:stat_compare_means) and GraphPad Prism 9. For analysis of the study population, Wilcoxon’s signed-rank and χ^2^ tests were used as indicated. Median values in each group for humoral, cellular, IL-10, and T cell subset analysis were compared by Kruskal-Wallis test (with Dunn’s post hoc multiple comparison test) or Wilcoxon’s signed-rank test. For pairwise analysis, Wilcoxon’s matched-pairs signed-rank test was performed. For correlation analysis, Spearman’s rank correlation coefficient (ρ) was calculated. Where applicable, statistical tests used and are indicated in the figure legends. *P* values of less than or equal to 0.05 were considered statistically significant. Categorized data with less than 3 independent samples were not included for statistical analysis. Data from flow cytometry was analyzed using FlowJo (BD).

### Study approval.

The study protocol was reviewed and approved by the SingHealth Centralised Institutional Review Board (Singapore) with CIRB reference 2021/2398. All donors provided written consent for enrollment.

## Author contributions

MQ, NLB, AB, and ES conceptualized and designed the experiments. MQ, SKH, WYW, and WN performed experiments for measuring humoral responses. MQ, HSK, and SH performed experiments for measuring cellular responses. MQ, NLB, AB, and ES analyzed the data. MQ and ES prepared the figures and tables. JXYS, JGHL, and ES acquired funding for the project. WPWC, MT, ES, JGHL, and TLA collected donor samples. The manuscript was prepared and edited by MQ, NLB, WYW, AB, and ES.

## Supplementary Material

Supplemental data

ICMJE disclosure forms

## Figures and Tables

**Table 2:**
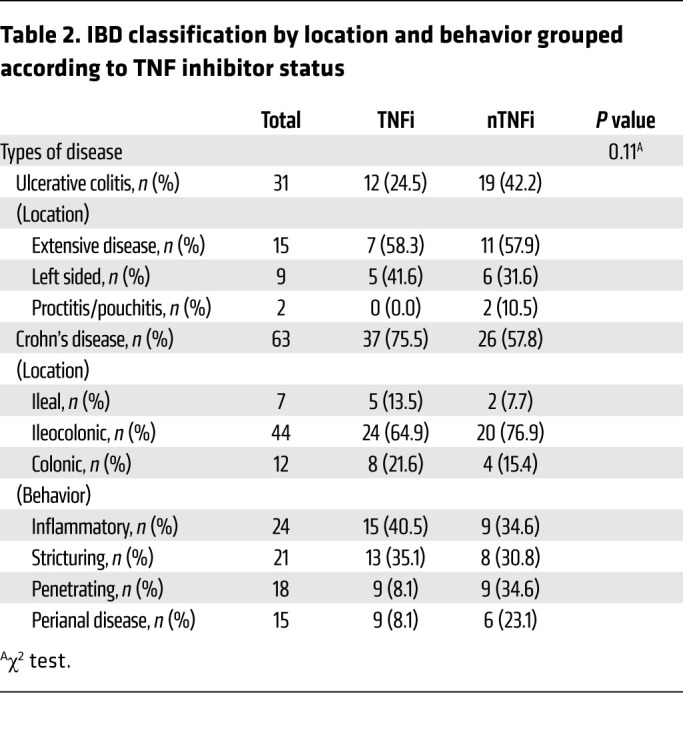
IBD classification by location and behavior grouped according to TNF inhibitor status

**Table 1:**
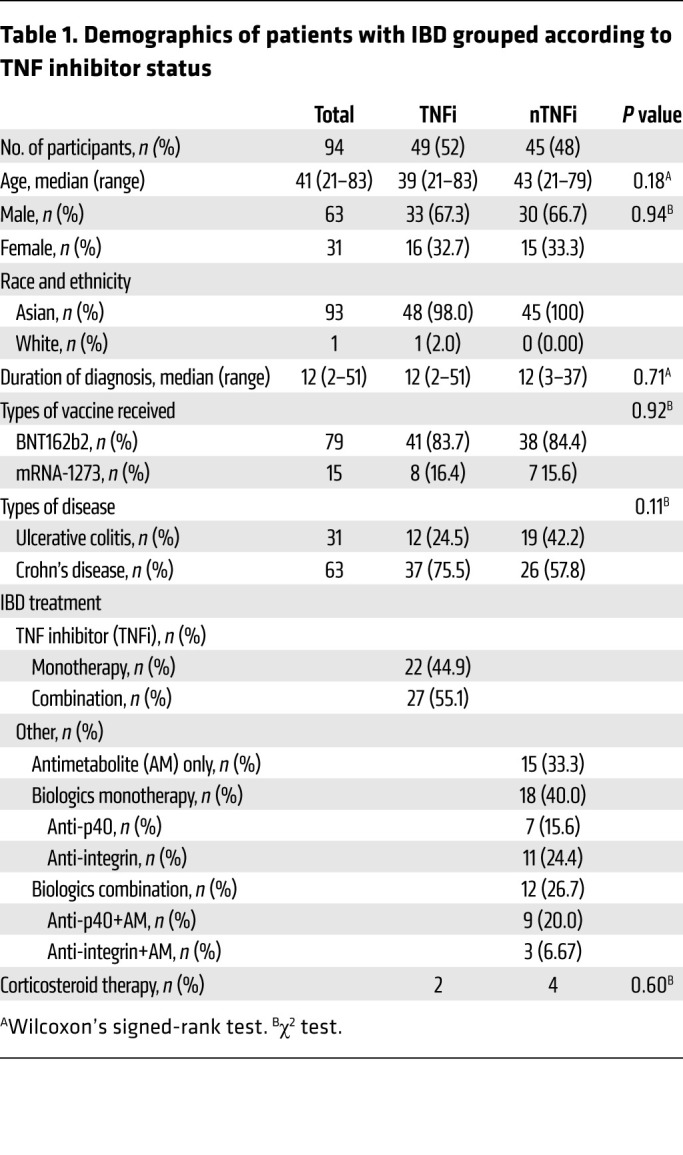
Demographics of patients with IBD grouped according to TNF inhibitor status

## Version 1 05/10/2022

In-Press Preview

## Version 2 06/15/2022

Electronic publication
