# Supplementary material for: Favorable vaccine-induced SARS-CoV-2–specific T cell response profile in patients undergoing immune-modifying therapies
Source: J Clin Invest. 2022 Jun 15;132(12):e159500. doi: 10.1172/JCI159500 (PMC9197512; doi:10.1172/JCI159500)
Supplement: Supplemental data [file jci-132-159500-s206.pdf]

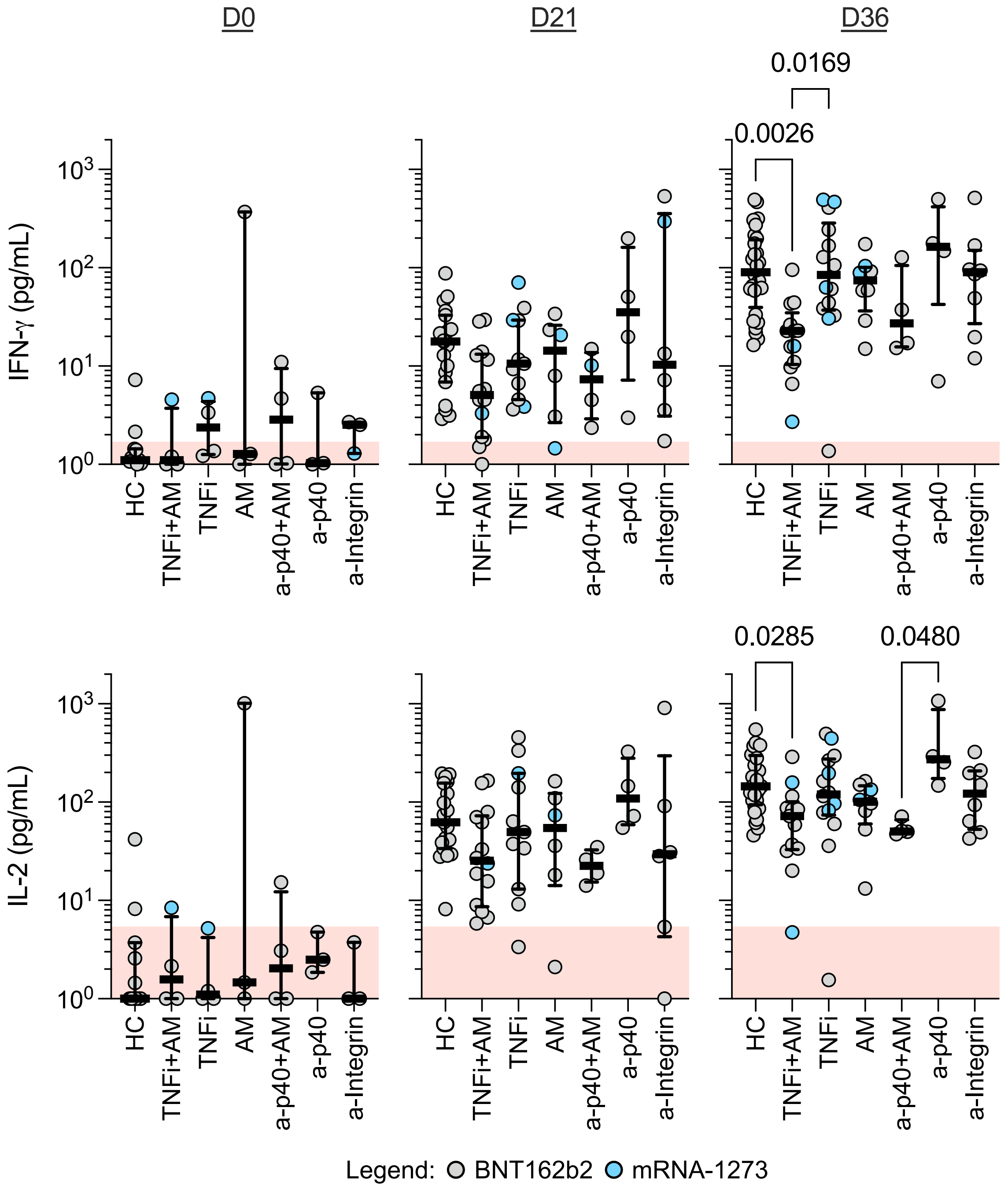

Figure S1. Early T cell responses in patients under different immunotherapies. (**A**) Dot plots with median line (middle bar) and interquartile range (whiskers) of quantified IFN-γ or IL-2 concentrations (pg/mL) from S pool-stimulated whole blood supernatants of HC and IBD patients grouped by treatment and faceted according to timepoint. Shaded red region denotes the area under the threshold for a positive test. Statistical analyses were performed by Kruskal-Wallis and Dunn's test with p values shown above the comparison lines when significant (α=0.05). For D 0: HC (n=10), TNFi+AM (n=4), TNFi (n=4), AM (n=3), a-p40+AM (n=4), a-p40 (n=3), a-Integrin (n=3). For D 21: HC (n=19), TNFi+AM (n=14), TNFi (n=11), AM (n=6), a-p40+AM (n=4), a-p40 (n=4), a-Integrin (n=6). For D 36: HC (n=28), TNFi+AM (n=14), TNFi (n=14), AM (n=8), a-p40+AM (n=5), a-p40 (n=4), a-Integrin (n=8).

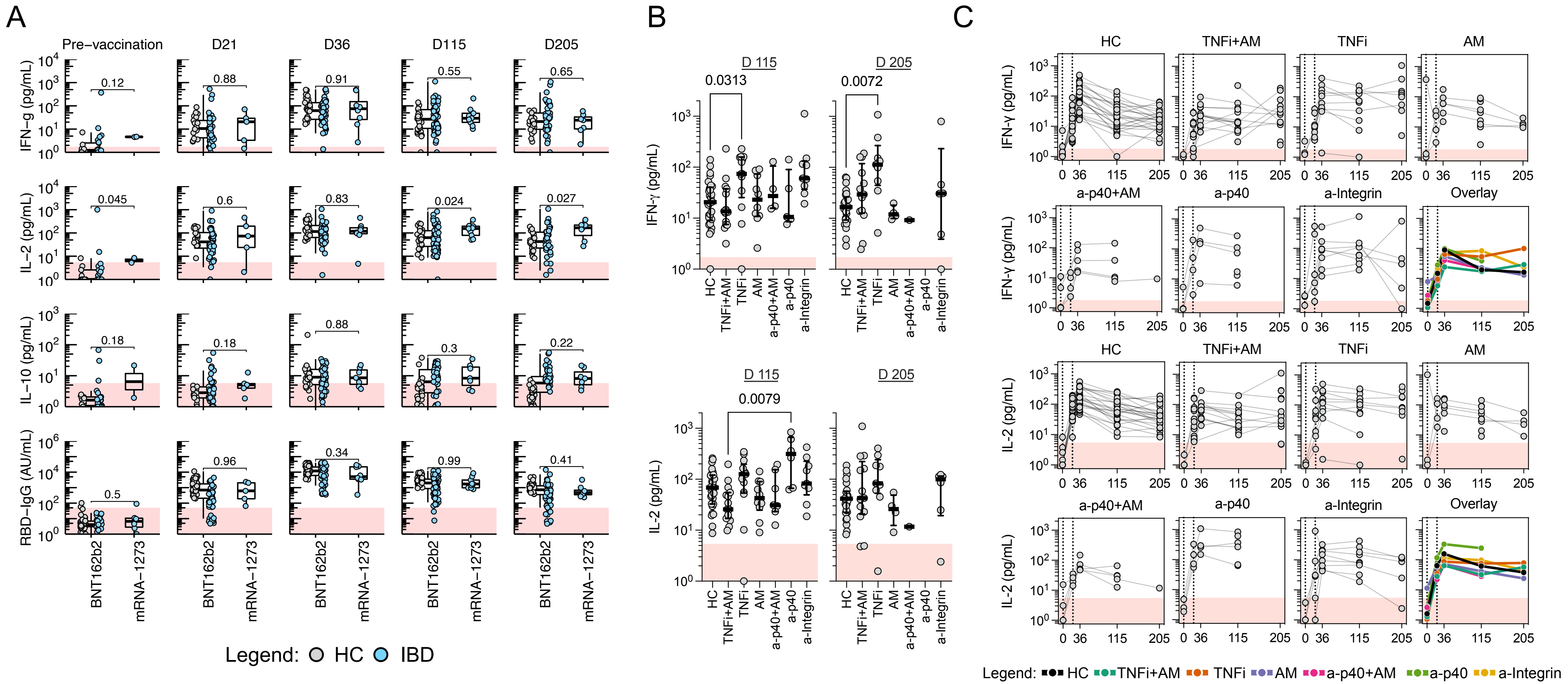

Figure S2. Vaccine-induced T cell responses based on mRNA vaccine administered. (**A**) Box and whisker plots with median line. Statistical analyses were performed by Wilcoxon signed rank test with p values indicated above the comparison line when significant (α=0.05). For RBD-IgG D0-D205: BNT162b2 (n=86, 91, 96, 110, 98), mRNA-1273 (n=6, 5, 8, 9, 8). For RBD-IgG D0-D205: BNT162b2 (n=86, 91, 96, 110, 98), mRNA-1273 (n=6, 5, 8, 9, 8). For IFN-γ/IL-2 D0-D205: BNT162b2 (n=29, 59, 73, 87, 61), mRNA-1273 (n=2, 5, 8, 9, 7). For IL-10 D0-D205: BNT162b2 (n=29, 59, 72, 66, 58), mRNA-1273 (n=2, 5, 7, 7, 7). (**B**) Dot plots with median line (middle bar) and interquartile range (whiskers) of quantified IFN-γ or IL-2 concentrations (pg/mL) from S pool-stimulated whole blood supernatants of HC and IBD patients without mRNA-1273 vaccinees grouped by treatment 3 and 6 months after completing their two-dose vaccination. Statistical analyses were performed by Kruskal-Wallis and Dunn's test with p values shown above the comparison lines when significant (α=0.05). For D 115: HC (n=29), TNFi+AM (n=15), TNFi (n=13), AM (n=10), a-p40+AM (n=5), a-p40 (n=6), a-Integrin (n=9). For D 205: HC (n=25), TNFi+AM (n=13), TNFi (n=9), AM (n=4), a-p40+AM (n=1), a-p40 (n=6), a-Integrin (n=9). **(C)** Quantified IFN-γ or IL-2 concentrations (pg/mL) plotted against time of HC and IBD patients grouped by treatment without mRNA-1273 vaccinees. Datapoints originating from the same participant are connected by gray lines. Data is summarized in the 'Overlay' plot with lines connecting the geometric means of each group at each sampling interval. (**A-C**) Shaded red regions denote the area under the threshold for a positive test.

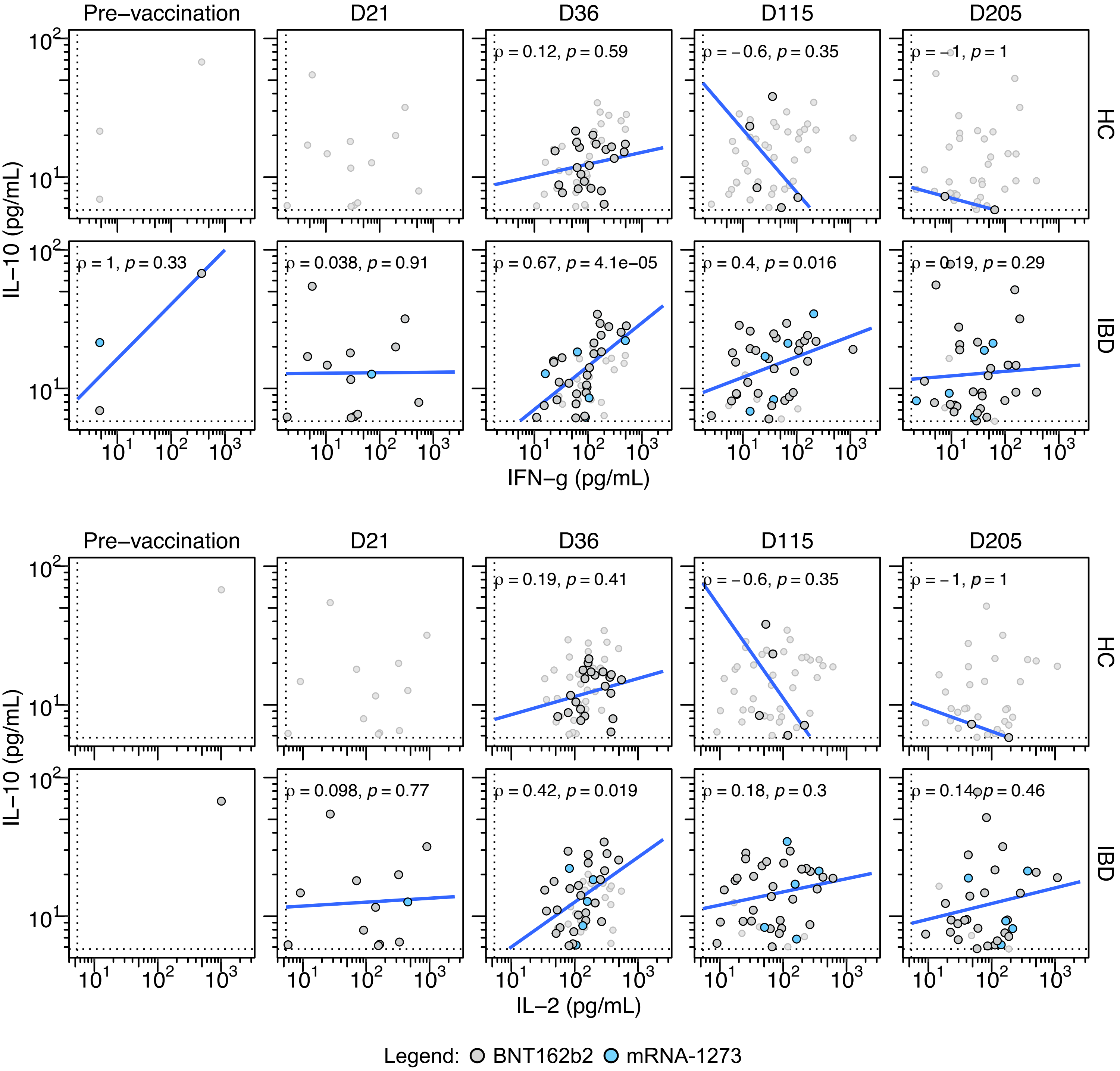

Figure S3. 2-D Correlation plots between peptide-induced IFN-γ/IL-2 and IL-10 from whole blood cytokine release assay between the HC and IBD cohorts. Data from each donor are faceted by cohort (rows) and timepoint (columns). All points from the same timepoint are displayed in each plot, which points originating from the designated cohort (row titles) highlighted. Datapoints below the threshold defined for each analyte (broken horizontal and vertical lines) for a positive response have been excluded from the nonparametric Spearman correlation analysis. Spearman's rho (ρ) and p values are inscribed into the plots when calculations are appropriate. Regression lines are drawn in blue (linear regression) used for visualisation purposes.

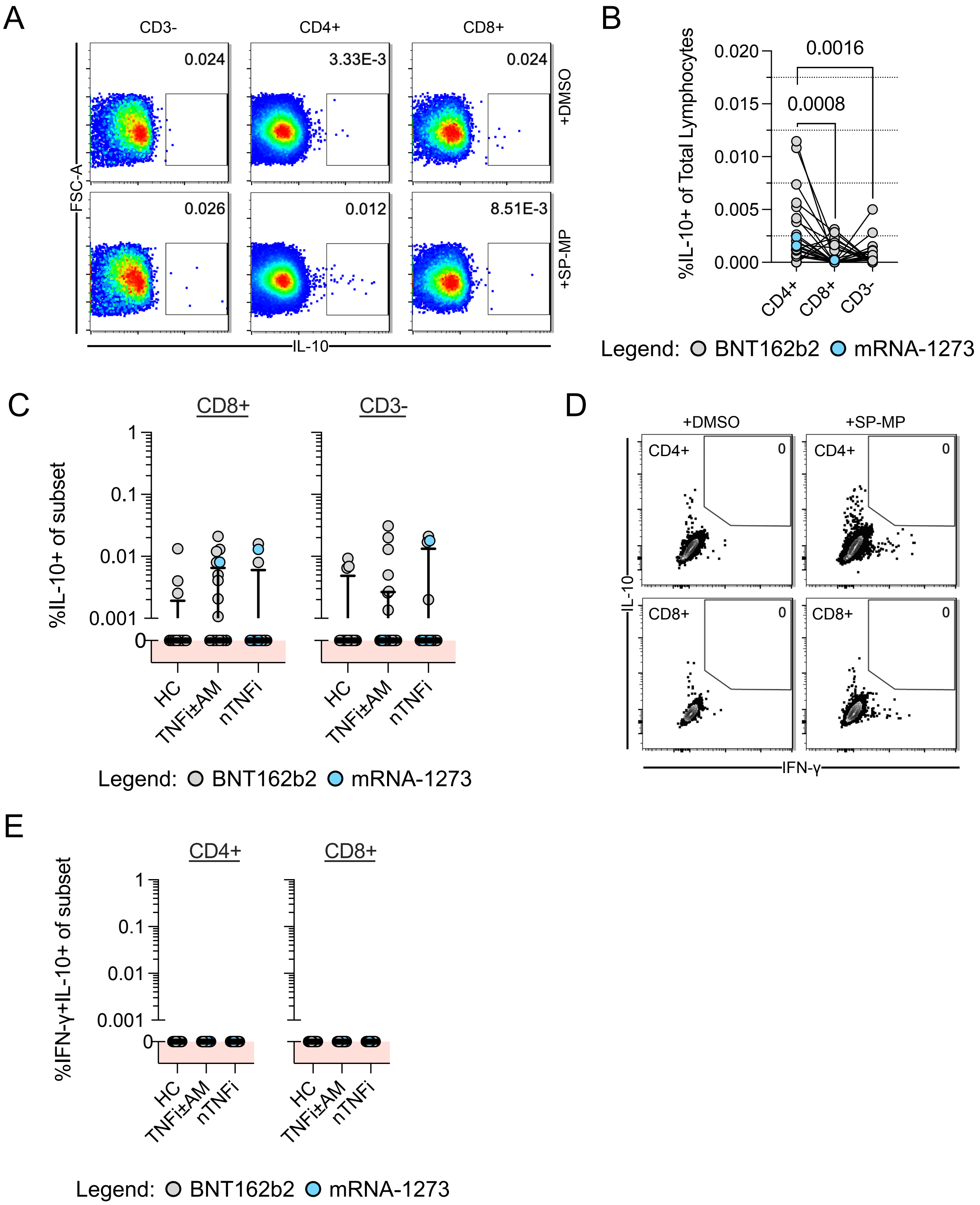

Figure S4. IL-10 production in peripheral blood lymphocytes. (**A** and **D**) Representative flow cytometry plots from intracellular cytokine staining to identify IL-10+ (**A**) or IFN-γ+ IL-10+ (**D**) T cell populations with or without stimulation with Spike peptides (drawn in gate). (**B**) Summary frequencies of paired background-subtracted IL-10+ frequencies identified from IBD patient PBMCs (n=30). Only samples with at least one subset of IL-10+ cells above background were included in the analysis. Statistical analysis was performed using multiple Wilcoxon matched-pairs signed rank test with p values indicated above the comparison line when significant (α=0.05). (**C** and **E**) Dot plots with median line (middle bar) and interquartile range (whiskers) of cellular frequencies of intracellular CD8+ or CD3- IL-10+ (**C**) and CD4+ or CD8+ IFN-γ+IL-10+ (**E**) cells from HC (n=12) or IBD donors on TNFi±AM (n=21) or nTNFi therapy (n=12). Statistical analyses were performed by Wilcoxon signed rank test with p values indicated above the comparison line when significant (α=0.05). Shaded red regions denote responses below background levels (denoted as 0).

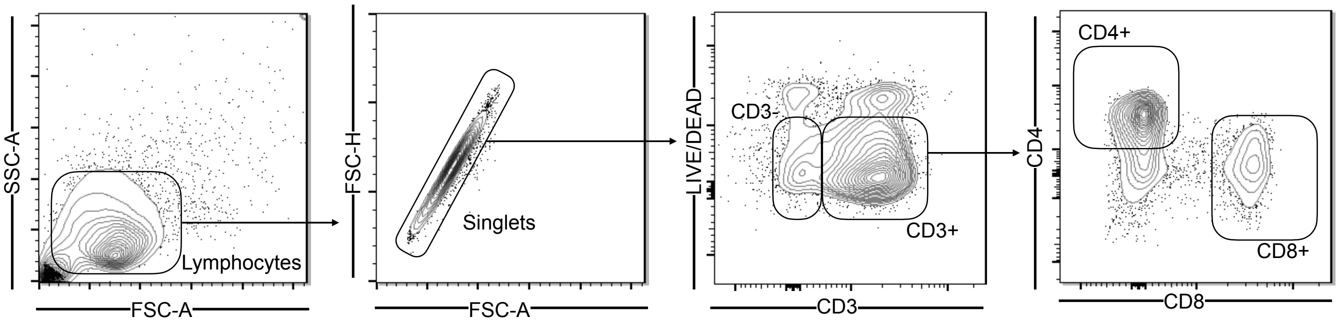

Figure S5. Representative gating strategy of T cell subsets for activation-induced markers assay/intracellular cytokine staining. Bivariate contour plots with outliers displaying a measured parameter obtained from flow cytometry indicated at the lower left corner of each plot. Gates are outlined in each plot. Similar gating strategies for T cell subsets are used for AIM and ICS assays.

Table S1. SARS-CoV-2 S pool peptide library

Peptide #	Peptide Sequence	aa
**1**	IRGWIFGTTLDSKTQ	101-115
**2**	FGTTLDSKTQSLLIV	106-120
**3**	CTFEYVSQPFLMDLE	166-180
**4**	VSQPFLMDLEGKQGN	171-185
**5**	TRFQTLLALHRSYLT	236-250
**6**	LLALHRSYLTPGDSS	241-255
**7**	RSYLTPGDSSSGWTA	246-260
**8**	CALDPLSETKCTLKS	291-305
**9**	LSETKCTLKSFTVEK	296-310
**10**	CTLKSFTVEKGIYQT	301-315
**11**	FTVEKGIYQTSNFRV	306-320
**12**	GIYQTSNFRVQPTES	311-325
**13**	SASFSTFKCYGVSPT	371-385
**14**	TFKCYGVSPTKLNDL	376-390
**15**	YNYKLPDDFTGCVIA	421-435
**16**	WNSNNLDSKVGGNYN	436-450
**17**	LDSKVGGNYNYLYRL	441-455
**18**	GGNYNYLYRLFRKSN	446-460
**19**	YLYRLFRKSNLKPFE	451-465
**20**	FRKSNLKPFERDIST	456-470
**21**	LKPFERDISTEIYQA	461-475
**22**	GPKKSTNLVKNKCVN	526-540
**23**	TNLVKNKCVNFNFNG	531-545
**24**	FNFNGLTGTGVLTES	541-555
**25**	LTGTGVLTESNKKFL	546-560
**26**	RAGCLIGAEHVNNSY	646-660
**27**	IGAEHVNNSYECDIP	651-665
**28**	SVASQSIIAYTMSLG	686-700
**29**	SIIAYTMSLGAENSV	691-705
**30**	TMSLGAENSVAYSNN	696-710
**31**	STECSNLLLQYGSFC	746-760
**32**	NLLLQYGSFCTQLNR	751-765
**33**	KNTQEVFAQVKQIYK	776-790
**34**	VFAQVKQIYKTPPIK	781-795

Peptide #	Peptide Sequence	aa
**35**	KQIYKTPPIKDFGGF	786-800
**36**	TPPIKDFGGFNFSQI	791-805
**37**	NFSQILPDPSKPSKR	801-815
**38**	AGFIKQYGDCLGDIA	831-845
**39**	QYGDCLGDIAARDLI	836-850
**40**	GAALQIPFAMQMAYR	891-905
**41**	QMAYRFNGIGVTQNV	901-915
**42**	FNGIGVTQNVLYENQ	906-920
**43**	DSLSSTASALGKLQD	936-950
**44**	TASALGKLQDVVNQN	941-955
**45**	AQALNTLVKQLSSNF	956-970
**46**	VLNDILSRLDKVEAE	976-990
**47**	LITGRLQSLQTYVTQ	996-1010
**48**	QLIRAAEIRASANLA	1011-1025
**49**	AEIRASANLAATKMS	1016-1030
**50**	APHGVVFLHVTYVPA	1056-1070
**51**	HWFVTQRNFYEPQII	1101-1115
**52**	KEIDRLNEVAKNLNE	1181-1195
**53**	LNEVAKNLNESLIDL	1186-1200
**54**	KNLNESLIDLQELGK	1191-1205
**55**	IWLGFIAGLIAIVMV	1216-1230

Table S2: SARS-CoV-2 SP-MP pool peptide library

Peptide #	Peptide Sequence	aa
**1**	MFVFLVLLPLVSSQC	1-15
**2**	VLLPLVSSQCVNLTT	6-20
**3**	VSSQCVNLTTRTQLP	11-25
**4**	VNLTTRTQLPPAYTN	16-30
**5**	RTQLPPAYTNSFTRG	21-35
**6**	PAYTNSFTRGVYYPD	26-40
**7**	SFTRGVYYPDKVFRS	31-45
**8**	VYYPDKVFRSSVLHS	36-50
**9**	KVFRSSVLHSTQDLF	41-55
**10**	SVLHSTQDLFLPFFS	46-60
**11**	TQDLFLPFFSNVTWF	51-65
**12**	LPFFSNVTWFHAIHV	56-70
**13**	NVTWFHAIHVSGTNG	61-75
**14**	HAIHVSGTNGTKRFD	66-80
**15**	SGTNGTKRFDNPVLP	71-85
**16**	TKRFDNPVLPFNDGV	76-90
**17**	NPVLPFNDGVYFAST	81-95
**18**	FNDGVYFASTEKSNI	86-100
**19**	YFASTEKSNIIRGWI	91-105
**20**	EKSNIIRGWIFGTTL	96-110
**21**	IRGWIFGTTLDSKTQ	101-115
**22**	FGTTLDSKTQSLLIV	106-120
**23**	DSKTQSLLIVNNATN	111-125
**24**	SLLIVNNATNVVIKV	116-130
**25**	NNATNVVIKVCEFQF	121-135
**26**	VVIKVCEFQFCNDPF	126-140
**27**	CEFQFCNDPFLGVYY	131-145
**28**	CNDPFLGVYYHKNNK	136-150
**29**	LGVYYHKNNKSWMES	141-155
**30**	HKNNKSWMESEFRVY	146-160
**31**	SWMESEFRVYSSANN	151-165
**32**	EFRVYSSANNCTFEY	156-170
**33**	SSANNCTFEYVSQPF	161-175
**34**	CTFEYVSQPFLMDLE	166-180

Peptide #	Peptide Sequence	aa
**35**	VSQPFLMDLEGKQGN	171-185
**36**	LMDLEGKQGNFKNLR	176-190
**37**	GKQGNFKNLREFVFK	181-195
**38**	FKNLREFVFKNIDGY	186-200
**39**	EFVFKNIDGYFKIYS	191-205
**40**	NIDGYFKIYSKHTPI	196-210
**41**	FKIYSKHTPINLVRD	201-215
**42**	KHTPINLVRDLPQGF	206-220
**43**	NLVRDLPQGFSALEP	211-225
**44**	LPQGFSALEPLVDLP	216-230
**45**	SALEPLVDLPIGINI	221-235
**46**	LVDLPIGINITRFQT	226-240
**47**	IGINITRFQTLLALH	231-245
**48**	TRFQTLLALHRSYLT	236-250
**49**	LLALHRSYLTPGDSS	241-255
**50**	RSYLTPGDSSSGWTA	246-260
**51**	PGDSSSGWTAGAAAY	251-265
**52**	SGWTAGAAAYYVGYL	256-270
**53**	GAAAYYVGYLQPRTF	261-275
**54**	YVGYLQPRTFLLKYN	266-280
**55**	QPRTFLLKYNENGTI	271-285
**56**	LLKYNENGTITDAVD	276-290
**57**	ENGTITDAVDCALDP	281-295
**58**	TDAVDCALDPLSETK	286-300
**59**	CALDPLSETKCTLKS	291-305
**60**	LSETKCTLKSFTVEK	296-310
**61**	CTLKSFTVEKGIYQT	301-315
**62**	FTVEKGIYQTSNFRV	306-320
**63**	GIYQTSNFRVQPTES	311-325
**64**	SNFRVQPTESIVRFP	316-330
**65**	QPTESIVRFPNITNL	321-335
**66**	IVRFPNITNLCPFGE	326-340
**67**	NITNLCPFGEVFNAT	331-345
**68**	CPFGEVFNATRFASV	336-350
**69**	VFNATRFASVYAWNR	341-355

Peptide #	Peptide Sequence	aa
**70**	RFASVYAWNRKRISN	346-360
**71**	YAWNRKRISNCVADY	351-365
**72**	KRISNCVADYSVLYN	356-370
**73**	CVADYSVLYNSASFS	361-375
**74**	SVLYNSASFSTFKCY	366-380
**75**	SASFSTFKCYGVSPT	371-385
**76**	TFKCYGVSPTKLNDL	376-390
**77**	GVSPTKLNDLCFTNV	381-395
**78**	KLNDLCFTNVYADSF	386-400
**79**	CFTNVYADSFVIRGD	391-405
**80**	YADSFVIRGDEVRQI	396-410
**81**	VIRGDEVRQIAPGQT	401-415
**82**	EVRQIAPGQTGKIAD	406-420
**83**	APGQTGKIADYNYKL	411-425
**84**	GKIADYNYKLPDDFT	416-430
**85**	YNYKLPDDFTGCVIA	421-435
**86**	PDDFTGCVIAWNSNN	426-440
**87**	GCVIAWNSNNLDSKV	431-445
**88**	WNSNNLDSKVGGNYN	436-450
**89**	LDSKVGGNYNYLYRL	441-455
**90**	GGNYNYLYRLFRKSN	446-460
**91**	YLYRLFRKSNLKPFE	451-465
**92**	FRKSNLKPFERDIST	456-470
**93**	LKPFERDISTEIYQA	461-475
**94**	RDISTEIYQAGSTPC	466-480
**95**	EIYQAGSTPCNGVEG	471-485
**96**	GSTPCNGVEGFNCYF	476-490
**97**	NGVEGFNCYFPLQSY	481-495
**98**	FNCYFPLQSYGFQPT	486-500
**99**	PLQSYGFQPTNGVGY	491-505
**100**	GFQPTNGVGYQPYRV	496-510
**101**	NGVGYQPYRVVVLSF	501-515
**102**	QPYRVVVLSFELLHA	506-520
**103**	VVLSFELLHAPATVC	511-525

Peptide #	Peptide Sequence	aa
**104**	ELLHAPATVCGPKKS	516-530
**105**	PATVCGPKKSTNLVK	521-535
**106**	GPKKSTNLVKNKCVN	526-540
**107**	TNLVKNKCVNFNFNG	531-545
**108**	NKCVNFNFNGLTGTG	536-550
**109**	FNFNGLTGTGVLTES	541-555
**110**	LTGTGVLTESNKKFL	546-560
**111**	VLTESNKKFLPFQQF	551-565
**112**	NKKFLPFQQFGRDIA	556-570
**113**	PFQQFGRDIADTTDA	561-575
**114**	GRDIADTTDAVRDPQ	566-580
**115**	DTTDAVRDPQTLEIL	571-585
**116**	VRDPQTLEILDITPC	576-590
**117**	TLEILDITPCSFGGV	581-595
**118**	DITPCSFGGVSVITP	586-600
**119**	SFGGVSVITPGTNTS	591-605
**120**	SVITPGTNTSNQVAV	596-610
**121**	GTNTSNQVAVLYQDV	601-615
**122**	NQVAVLYQDVNCTEV	606-620
**123**	LYQDVNCTEVPVAIH	611-625
**124**	NCTEVPVAIHADQLT	616-630
**125**	PVAIHADQLTPTWRV	621-635
**126**	ADQLTPTWRVYSTGS	626-640
**127**	PTWRVYSTGSNVFQT	631-645
**128**	YSTGSNVFQTRAGCL	636-650
**129**	NVFQTRAGCLIGAEH	641-655
**130**	RAGCLIGAEHVNNSY	646-660
**131**	IGAEHVNNSYECDIP	651-665
**132**	VNNSYECDIPIGAGI	656-670
**133**	ECDIPIGAGICASYQ	661-675
**134**	IGAGICASYQTQTNS	666-680
**135**	CASYQTQTNSPRRAR	671-685
**136**	TQTNSPRRARSVASQ	676-690
**137**	PRRARSVASQSIIAY	681-695

Peptide #	Peptide Sequence	aa
**138**	SVASQSIIAYTMSLG	686-700
**139**	SIIAYTMSLGAENSV	691-705
**140**	TMSLGAENSVAYSNN	696-710
**141**	AENSVAYSNNSIAIP	701-715
**142**	AYSNNSIAIPTNFTI	706-720
**143**	SIAIPTNFTISVTTE	711-725
**144**	TNFTISVTTEILPVS	716-730
**145**	SVTTEILPVSMTKTS	721-735
**146**	ILPVSMTKTSVDCTM	726-740
**147**	MTKTSVDCTMYICGD	731-745
**148**	VDCTMYICGDSTECS	736-750
**149**	YICGDSTECSNLLLQ	741-755
**150**	STECSNLLLQYGSFC	746-760
**151**	NLLLQYGSFCTQLNR	751-765
**152**	YGSFCTQLNRALTGI	756-770
**153**	TQLNRALTGIAVEQD	761-775
**154**	ALTGIAVEQDKNTQE	766-780
**155**	AVEQDKNTQEVFAQV	771-785
**156**	KNTQEVFAQVKQIYK	776-790
**157**	VFAQVKQIYKTPPIK	781-795
**158**	KQIYKTPPIKDFGGF	786-800
**159**	TPPIKDFGGFNFSQI	791-805
**160**	DFGGFNFSQILPDPS	796-810
**161**	NFSQILPDPSKPSKR	801-815
**162**	LPDPSKPSKRSFIED	806-820
**163**	KPSKRSFIEDLLFNK	811-825
**164**	SFIEDLLFNKVTLAD	816-830
**165**	LLFNKVTLADAGFIK	821-835
**166**	VTLADAGFIKQYGDC	826-840
**167**	AGFIKQYGDCLGDIA	831-845
**168**	QYGDCLGDIAARDLI	836-850
**169**	LGDIAARDLICAQKF	841-855
**170**	ARDLICAQKFNGLTV	846-860
**171**	CAQKFNGLTVLPPLL	851-865

Peptide #	Peptide Sequence	aa
**172**	NGLTVLPPLLTDEMI	856-870
**173**	LPPLLTDEMIAQYTS	861-875
**174**	TDEMIAQYTSALLAG	866-880
**175**	AQYTSALLAGTITSG	871-885
**176**	ALLAGTITSGWTFGA	876-890
**177**	TITSGWTFGAGAALQ	881-895
**178**	WTFGAGAALQIPFAM	886-900
**179**	GAALQIPFAMQMAYR	891-905
**180**	IPFAMQMAYRFNGIG	896-910
**181**	QMAYRFNGIGVTQNV	901-915
**182**	FNGIGVTQNVLYENQ	906-920
**183**	VTQNVLYENQKLIAN	911-925
**184**	LYENQKLIANQFNSA	916-930
**185**	KLIANQFNSAIGKIQ	921-935
**186**	QFNSAIGKIQDSLSS	926-940
**187**	IGKIQDSLSSTASAL	931-945
**188**	DSLSSTASALGKLQD	936-950
**189**	TASALGKLQDVVNQN	941-955
**190**	GKLQDVVNQNAQALN	946-960
**191**	VVNQNAQALNTLVKQ	951-965
**192**	AQALNTLVKQLSSNF	956-970
**193**	TLVKQLSSNFGAISS	961-975
**194**	LSSNFGAISSVLNDI	966-980
**195**	GAISSVLNDILSRLD	971-985
**196**	VLNDILSRLDKVEAE	976-990
**197**	LSRLDKVEAEVQIDR	981-995
**198**	KVEAEVQIDRLITGR	986-1000
**199**	VQIDRLITGRLQSLQ	991-1005
**200**	LITGRLQSLQTYVTQ	996-1010
**201**	LQSLQTYVTQQLIRA	1001-1015
**202**	TYVTQQLIRAAEIRA	1006-1020
**203**	QLIRAAEIRASANLA	1011-1025
**204**	AEIRASANLAATKMS	1016-1030
**205**	SANLAATKMSECVLG	1021-1035

Peptide #	Peptide Sequence	aa
**206**	ATKMSECVLGQSKRV	1026-1040
**207**	ECVLGQSKRVDFCGK	1031-1045
**208**	QSKRVDFCGKGYHLM	1036-1050
**209**	DFCGKGYHLMSFPQS	1041-1055
**210**	GYHLMSFPQSAPHGV	1046-1060
**211**	SFPQSAPHGVVFLHV	1051-1065
**212**	APHGVVFLHVTYVPA	1056-1070
**213**	VFLHVTYVPAQEKNF	1061-1075
**214**	TYVPAQEKNFTTAPA	1066-1080
**215**	QEKNFTTAPAICHDG	1071-1085
**216**	TTAPAICHDGKAHFP	1076-1090
**217**	ICHDGKAHFPREGVF	1081-1095
**218**	KAHFPREGVFVSNGT	1086-1100
**219**	REGVFVSNGTHWFVT	1091-1105
**220**	VSNGTHWFVTQRNFY	1096-1110
**221**	HWFVTQRNFYEPQII	1101-1115
**222**	QRNFYEPQIITTDNT	1106-1120
**223**	EPQIITTDNTFVSGN	1111-1125
**224**	TTDNTFVSGNCDVVI	1116-1130
**225**	FVSGNCDVVIGIVNN	1121-1135
**226**	CDVVIGIVNNTVYDP	1126-1140
**227**	GIVNNTVYDPLQPEL	1131-1145
**228**	TVYDPLQPELDSFKE	1136-1150
**229**	LQPELDSFKEELDKY	1141-1155
**230**	DSFKEELDKYFKNHT	1146-1160
**231**	ELDKYFKNHTSPDVD	1151-1165
**232**	FKNHTSPDVDLGDIS	1156-1170
**233**	SPDVDLGDISGINAS	1161-1175
**234**	LGDISGINASVVNIQ	1166-1180
**235**	GINASVVNIQKEIDR	1171-1185
**236**	VVNIQKEIDRLNEVA	1176-1190
**237**	KEIDRLNEVAKNLNE	1181-1195
**238**	LNEVAKNLNESLIDL	1186-1200
**239**	KNLNESLIDLQELGK	1191-1205

Peptide #	Peptide Sequence	aa
**240**	SLIDLQELGKYEQYI	1196-1210
**241**	QELGKYEQYIKWPWY	1201-1215
**242**	YEQYIKWPWYIWLGF	1206-1220
**243**	KWPWYIWLGFIAGLI	1211-1225
**244**	IWLGFIAGLIAIVMV	1216-1230
**245**	IAGLIAIVMVTIMLC	1221-1235
**246**	AIVMVTIMLCCMTSC	1226-1240
**247**	TIMLCCMTSCCSCLK	1231-1245
**248**	CMTSCCSCLKGCCSC	1236-1250
**249**	CSCLKGCCSCGSCCK	1241-1255
**250**	GCCSCGSCCKFDEDD	1246-1260
**251**	GSCCKFDEDDSEPVL	1251-1265
**252**	FDEDDSEPVLKGVKL	1256-1270
**253**	SEPVLKGVKLHYT	1261-1273

Table S3. SARS-CoV-2 Spike Hotspot-Ancestral and Hotspot-Omicron peptide libraries used to study responses to the Omicron variant (BA.1)

Peptide #	Spike Hotspot-Ancestral	Spike Hotspot-Omicron	aa
**1**	LPFFSNVTWFHAIHV	LPFFSNVTWFHVISG	56-70
**2**	NVTWFHAIHVSGTNG	NVTWFHVISGTNGTK	61-75
**3**	HAIHVSGTNGTKRFD	HVISGTNGTKRFDNP	66-80
**4**	FNDGVYFASTEKSNI	FNDGVYFASIEKSNI	86-100
**5**	YFASTEKSNIIRGWI	YFASIEKSNIIRGWI	91-105
**6**	CEFQFCNDPFLGVYY	CEFQFCNDPFLDHKN	131-145
**7**	CNDPFLGVYYHKNNK	CNDPFLDHKNNKSWM	136-150
**8**	LGVYYHKNNKSWMES	LDHKNNKSWMESEFR	141-155
**9**	FKIYSKHTPINLVRD	FKIYSKHTPIIVEPE	201-215
**10**	KHTPINLVRDLPQGF	KHTPIIVEPERDLPQ	206-220
**11**	NLVRDLPQGFSALEP	IVEPERDLPQGFSAL	211-225
**12**	IVRFPNITNLCPFGE	IVRFPNITNLCPFDE	326-340
**13**	NITNLCPFGEVFNAT	NITNLCPFDEVFNAT	331-345
**14**	CPFGEVFNATRFASV	CPFDEVFNATRFASV	336-350
**15**	CVADYSVLYNSASFS	CVADYSVLYNLAPFF	361-375
**16**	SVLYNSASFSTFKCY	SVLYNLAPFFTFKCY	366-380
**17**	SASFSTFKCYGVSPT	LAPFFTFKCYGVSPT	371-385
**18**	EVRQIAPGQTGKIAD	EVRQIAPGQTGNIAD	406-420
**19**	APGQTGKIADYNYKL	APGQTGNIADYNYKL	411-425
**20**	GKIADYNYKLPDDFT	GNIADYNYKLPDDFT	416-430
**21**	PDDFTGCVIAWNSNN	PDDFTGCVIAWNSNK	426-440
**22**	GCVIAWNSNNLDSKV	GCVIAWNSNKLDSKV	431-445
**23**	WNSNNLDSKVGGNYN	WNSNKLDSKVSGNYN	436-450
**24**	LDSKVGGNYNYLYRL	LDSKVSGNYNYLYRL	441-455
**25**	GGNYNYLYRLFRKSN	SGNYNYLYRLFRKSN	446-460
**26**	RDISTEIYQAGSTPC	RDISTEIYQAGNKPC	466-480
**27**	EIYQAGSTPCNGVEG	EIYQAGNKPCNGVAG	471-485
**28**	GSTPCNGVEGFNCYF	GNKPCNGVAGFNCYF	476-490
**29**	NGVEGFNCYFPLQSY	NGVAGFNCYFPLRSY	481-495
**30**	FNCYFPLQSYGFQPT	FNCYFPLRSYSFRPT	486-500
**31**	PLQSYGFQPTNGVGY	PLRSYSFRPTYGVGH	491-505
**32**	GFQPTNGVGYQPYRV	SFRPTYGVGHQPYRV	496-510
**33**	NGVGYQPYRVVVLSF	YGVGHQPYRVVVLSF	501-515
**34**	NKCVNFNFNGLTGTG	NKCVNFNFNGLKGTG	536-550
**35**	FNFNGLTGTGVLTES	FNFNGLKGTGVLTES	541-555
**36**	LTGTGVLTESNKKFL	LKGTGVLTESNKKFL	546-560

Peptide #	Spike Hotspot-Ancestral	Spike Hotspot-Omicron	aa
**37**	GTNTSNQVAVLYQDV	GTNTSNQVAVLYQGV	601-615
**38**	NQVAVLYQDVNCTEV	NQVAVLYQGVNCTEV	606-620
**39**	LYQDVNCTEVPVAIH	LYQGVNCTEVPVAIH	611-625
**40**	NVFQTRAGCLIGAEH	NVFQTRAGCLIGAEY	641-655
**41**	RAGCLIGAEHVNNSY	RAGCLIGAEYVNNSY	646-660
**42**	IGAEHVNNSYECDIP	IGAEYVNNSYECDIP	651-665
**43**	IGAGICASYQTQTNS	IGAGICASYQTQTKS	666-680
**44**	CASYQTQTNSPRRAR	CASYQTQTKSHRRAR	671-685
**45**	TQTNSPRRARSVASQ	TQTKSHRRARSVASQ	676-690
**46**	PRRARSVASQSIIAY	HRRARSVASQSIIAY	681-695
**47**	SIIAYTMSLGAENSV	SIIAYTMSLGVENSV	691-705
**48**	TMSLGAENSVAYSNN	TMSLGVENSVAYSNN	696-710
**49**	AENSVAYSNNSIAIP	VENSVAYSNNSIAIP	701-715
**50**	NLLLQYGSFCTQLNR	NLLLQYGSFCTQLKR	751-765
**51**	YGSFCTQLNRALTGI	YGSFCTQLKRALTGI	756-770
**52**	TQLNRALTGIAVEQD	TQLKRALTGIAVEQD	761-775
**53**	KQIYKTPPIKDFGGF	KQIYKTPPIKYFGGF	786-800
**54**	TPPIKDFGGFNFSQI	TPPIKYFGGFNFSQI	791-805
**55**	DFGGFNFSQILPDPS	YFGGFNFSQILPDPS	796-810
**56**	ARDLICAQKFNGLTV	ARDLICAQKFKGLTV	846-860
**57**	CAQKFNGLTVLPPLL	CAQKFKGLTVLPPLL	851-865
**58**	NGLTVLPPLLTDEMI	KGLTVLPPLLTDEMI	856-870
**59**	TASALGKLQDVVNQN	TASALGKLQDVVNHN	941-955
**60**	GKLQDVVNQNAQALN	GKLQDVVNHNAQALN	946-960
**61**	VVNQNAQALNTLVKQ	VVNHNAQALNTLVKQ	951-965
**62**	AQALNTLVKQLSSNF	AQALNTLVKQLSSKF	956-970
**63**	TLVKQLSSNFGAISS	TLVKQLSSKFGAISS	961-975
**64**	LSSNFGAISSVLNDI	LSSKFGAISSVLNDI	966-980
**65**	GAISSVLNDILSRLD	GAISSVLNDIFSRLD	971-985
**66**	VLNDILSRLDKVEAE	VLNDIFSRLDKVEAE	976-990
**67**	LSRLDKVEAEVQIDR	FSRLDKVEAEVQIDR	981-995

Table S4. List of labelling reagents used for flow cytometry

Target	Organism	Color	Clone	Label	RRID/Cat. no	Dilution (in 100µL)
CD3	anti-Human	BV650	SP34-2	BD Horizon™	AB_2738486	2.5
CD4	anti-Human	AF700	RPA-T4	Biolegend	AB_493743	2.5
CD8	anti-Human	V500	RPA-T8	BD Horizon™	AB_1937333	0.5
CD69	anti-Human	PE/Dazzle594	FN50	Biolegend	AB_2564276	2
CD134/OX40	anti-Human	PerCP-Cy5.5	Ber-ACT35	Biolegend	AB_10720986	2
CD137/4-1BB	anti-Human	PE-Cy5	4B4-1	BD Horizon™	AB_394067	5
IL-10	anti-Human	BV421	JES3-9D7	Biolegend	AB_10896947	6
IFN-gamma	anti-Human	PE-Cy7	4S.B3	eBioscience™	AB_1659719	6
IL-2	anti-Human	PerCP-Cy5.5	MQ1-17H12	BD Pharmingen™	AB_1727543	5
LIVE/DEAD™ Fixable Yellow Dead Cell Stain Kit (405nm excitation)				ThermoFisher	Cat. L34959	0.1
